# SARS-CoV2 infection in whole lung primarily targets macrophages that display subset-specific responses

**DOI:** 10.1007/s00018-024-05322-z

**Published:** 2024-08-15

**Authors:** Thien-Phong Vu Manh, Carla Gouin, Julien De Wolf, Luc Jouneau, Florentina Pascale, Claudia Bevilacqua, Meriadeg Ar Gouilh, Bruno Da Costa, Christophe Chevalier, Matthieu Glorion, Laurent Hannouche, Céline Urien, Jérôme Estephan, Antoine Magnan, Morgan Le Guen, Quentin Marquant, Delphyne Descamps, Marc Dalod, Isabelle Schwartz-Cornil, Edouard Sage

**Affiliations:** 1 Aix-Marseille University, CNRS, INSERM, CIML, Centre d′Immunologie de Marseille-Luminy, Turing Center for Living Systems, 13009 Marseille, France; 2 Université Paris-Saclay, INRAE, UVSQ, VIM, 78350 Jouy-en-Josas, France; 3 Department of Thoracic Surgery and Lung Transplantation, Foch Hospital, 92150 Suresnes, France; 4 Université Paris-Saclay, INRAE, UVSQ, BREED, 78350 Jouy-en-Josas, France; 5 Université Paris-Saclay, INRAE, AgroParisTech, GABI, 78350 Jouy-en-Josas, France; 6 Department of Pulmonology, Foch Hospital, 92150 Suresnes, France; 7 Department of Anesthesiology, Foch Hospital, 92150 Suresnes, France; 8 Department of Virology, Univ Caen Normandie, Dynamicure INSERM UMR 1311, CHU Caen, 14000 Caen, France; 9 Delegation to Clinical Research and Innovation, Foch Hospital, 92150 Suresnes, France

**Keywords:** Ex vivo lung perfusion, Viral nebulization, 10X genomics, Azimuth software

## Abstract

Deciphering the initial steps of SARS-CoV-2 infection, that influence COVID-19 outcomes, is challenging because animal models do not always reproduce human biological processes and in vitro systems do not recapitulate the histoarchitecture and cellular composition of respiratory tissues. To address this, we developed an innovative ex vivo model of whole human lung infection with SARS-CoV-2, leveraging a lung transplantation technique. Through single-cell RNA-seq, we identified that alveolar and monocyte-derived macrophages (AMs and MoMacs) were initial targets of the virus. Exposure of isolated lung AMs, MoMacs, classical monocytes and non-classical monocytes (ncMos) to SARS-CoV-2 variants revealed that while all subsets responded, MoMacs produced higher levels of inflammatory cytokines than AMs, and ncMos contributed the least. A Wuhan lineage appeared to be more potent than a D614G virus, in a dose-dependent manner. Amidst the ambiguity in the literature regarding the initial SARS-CoV-2 cell target, our study reveals that AMs and MoMacs are dominant primary entry points for the virus, and suggests that their responses may conduct subsequent injury, depending on their abundance, the viral strain and dose. Interfering on virus interaction with lung macrophages should be considered in prophylactic strategies.

**Supplementary Information:**

The online version contains supplementary material available at 10.1007/s00018-024-05322-z.

## Introduction

The SARS-CoV-2 virus is responsible for a large variety of clinical manifestations affecting the respiratory tract, going from a- or pauci-symptomatic state, to a pneumonia and an acute respiratory distress syndrome. In order to develop effective intervention strategies, several independent studies investigated the precise cell types targeted by the virus, from blood and broncho-alveolar lavages (BAL) at different stages of advanced diseases [1] and from lungs at autopsy [2]. In the BAL, SARS-CoV-2 was found associated with epithelial cells and macrophages [1], and in the lung of deceased patients, with mononuclear phagocytic cells, endothelial cells, pneumocytes and airway cells [2–4].

Among the cells reported to be targeted in these studies, the lung monocytes/macrophage compartment is a heterogeneous population of cell types that remains incompletely characterized. In the mouse model and in human, lung macrophages include two main cell types, named alveolar macrophages (AMs) and interstitial macrophages (IMs). The AMs are the guardians of the air-space homeostasis, through their catabolic, immunomodulatory and repair activities and they self-renew from local progenitors seeded during the fetal life or also differentiate postnatally from monocytes (Mos), see for a recent review [5]. IMs have been far less studied; in the mouse, they include distinct subtypes that occupy different parenchymal niches, such as the bronchial wall underneath epithelial cells [6, 7], the vicinity of nerves or blood vessels [8] or even the airspace upon stimulation [9]. In human, IMs were dominantly found in the alveolar septa [10]. Importantly, both AMs and IMs have been shown to be able to capture incoming pathogens from the lumen, such as in the case of Escherichia Coli and zymosan particles [7]. When studied side-by-side, AMs and IMs appear functionally different depending on contexts, AMs tending to be more phagocytic, and IMs more anti-inflammatory upon allergen sensitization [5]. Several studies support that IMs derive from blood monocytes [7–9, 11] and they are regarded as homologues of the so-called monocyte-derived macrophages (MoMacs) from the skin, heart and gut [7, 11]. In addition, AMs and IMs can be distinguished based on transcriptomic signatures and cell surface profiling as AMs and MoMacs respectively, a designation that we will use in this paper [7, 11]. Besides, human lung also contains Mos in the airspace and in the vascular compartment even after extensive lavage [12]. Human Mos include 3 different subsets, i.e. the classical CD14^pos^CD16^low^ monocytes (cMos) recruited to inflammatory sites, the non-classical CD14^low^CD16^pos^ (ncMos) also called patrolling monocytes which control endothelial integrity, and the intermediate CD14^pos^CD16^pos^ monocytes whose role is still unclear [13, 14]. During SARS-CoV-2 infection, these different monocyte/macrophage cell types (AMs, IMs alias MoMacs, cMos and ncMos) are massively perturbed, to extents that strongly correlate with disease severity [15–20]. However, these perturbations have only been studied in advanced disease. The initial response of the different lung monocyte/macrophage subsets has not been investigated comparatively.

The initial interaction of respiratory tract cells with pathogens conditions the balance between viral replication, effective innate host defense, and the development of uncontrolled inflammatory amplification. Indeed, in SARS-CoV-2 physiopathology, the initial innate response can promote the expression of Angiotensin Converting Enzyme 2 (ACE2) and Fc receptors on macrophages as well as induce an inflammatory M1 phenotype, all of which are associated with subsequent disease severity [21–24]. Several in vitro models have been established to mimic the initial interaction between lung cell types and SARS-CoV-2, mainly using explant-based techniques that generated different types of results: some reported that only epithelial cells were infected [25–27], others that both epithelial cells and macrophages were equally infected [28, 29], and finally that mainly alveolar macrophages were associated with the virus [30]. These contrasting results could be due to biases in the various tissue-culture conditions; indeed, none of these systems consider the spatial tissue architecture and anatomical constraints that play a crucial role in determining how different cell types interact with viruses.

In this study, we used a whole human lung maintained alive ex vivo for 10 h, based on a technique used in lung transplantation (ex vivo lung perfusion or EVLP), to analyze the first steps of SARS-CoV-2 infection in the lung. Human lungs declined for transplantation were perfused and ventilated at 37 °C and infected with SARS-CoV-2 using nebulization of the Wuhan lineage and D614G variants. The virus was found to be predominantly associated with AMs and MoMacs. Then we exposed to SARS-CoV-2, AMs, MoMacs, cMos and ncMos that were freshly isolated from human lungs, and we compared side-by-side their inflammatory responses. In a context of unclear knowledge regarding the initial cell targets of SARS-CoV-2 in the lung, these results obtained with human lungs clarify that both AMs and MoMacs are primary targets of SARS-CoV-2 and reveal that both produce cytokines and chemokines upon viral exposure, with MoMacs being the strongest responder cell type.

## Material and methods

### Cell line

Vero cells (E6 lineage, African Green monkey kidney epithelial cells) were obtained from the American Type Culture Collection (referred as VERO C1008 [Vero 76, clone E6, Vero E6], CRL-1586). Vero E6 cells were cultured in Dulbecco’s minimal essential medium (DMEM, Eurobio Scientific) supplemented with 5% fetal calf serum (FCS, Eurobio Scientific), 100 IU/ml penicillin, 100 μg/ml streptomycin at 37 °C.

### Study design

**Fig. 1:**
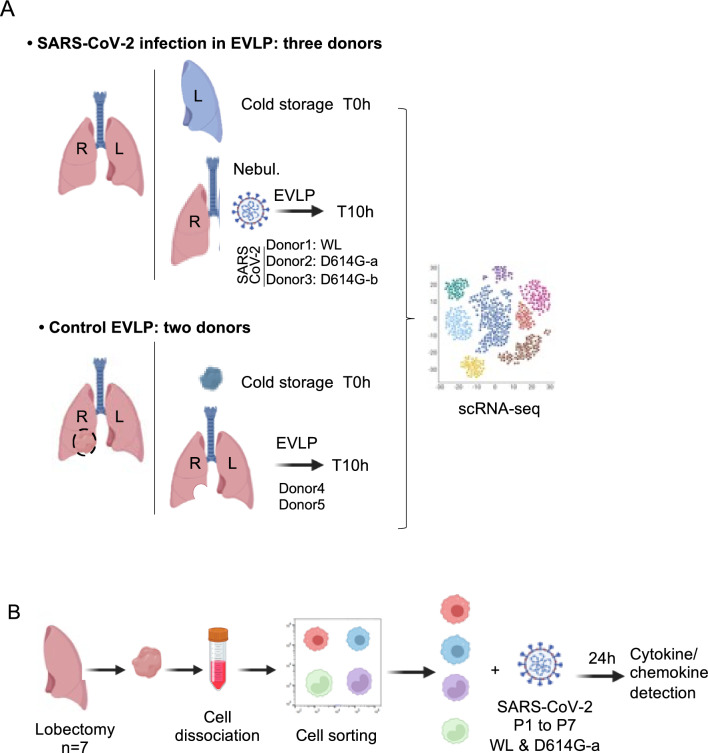
Experimental scheme. **A** The EVLP part of the study (see Methods). Three right lungs (Additional file 1-Anamnesis), processed to EVLP for 10 h, were infected with SARS-CoV-2 using a nebulizer at the onset of EVLP. Three different viruses were used: WL for donor 1 (1.2 × 10^8^ PFUs), D614G-a for donor 2 (3.3 × 10^7^ PFUs), D614G-b for donor 3 (10^7^ PFUs). A lung biopsy from the left lung was taken at 0 h and placed in hypothermosol at 4 °C. Another lung biopsy was taken at 10 h. Two control lungs were processed to EVLP for 10 h and the sampling was performed similarly. Lung cells were isolated from the EVLP biopsies collected at 0 h and 10 h and subjected to scRNA-seq. In the case of donor 2 and 3, additional samples with HLA-DR-enriched cells were subjected to scRNA-seq. **B** The isolated lung monocyte/macrophage part of the study (see Methods). Lung tissue samples were obtained from seven patients undergoing surgical resection for lung carcinoma (Additional file 1-Anamnese). Lung cells were isolated, labelled with mAbs for monocyte/macrophage identification, and sorted with a CytoFLEX sorter. The 4 purified subsets (AM, MoMacs, cMos, ncMos) were exposed to SARS-CoV-2 WL or D614G-a virus at 0.1 and 0.001 MOI for 24 h and the supernatants were assayed for cytokine detection using a human ProcartaPlexTM Mix&Match 12-plex. Created with Biorender.com

The EVLP part of the study (Fig. 1A) was approved by the Agence de la Biomédecine and by the French “Ministère de l’Enseignement Supérieur et de la Recherche, Direction Générale de la Recherche et de l’Innovation” under the number 2020–007, as well as by the ethic committee of the Foch Hospital (IRB00012437). The five donor lungs used in this study were from donation after brain death and were determined to be unsuitable for transplantation (Additional file 1. Anamnesis). Donor lung retrieval was carried out according to current clinical practice using Perfadex (Xvivo Perfusion, Göteborg, Sweden) flush preservation. After transportation at 4 °C, lungs were processed to EVLP that was conducted according to the Toronto protocol for 10 h [31] in the BSL3 facilities of the Molecular Virology and Immunology laboratory in Jouy en Josas in the case of the SARS-CoV-2 infection in EVLP, and at the Foch Hospital, Suresnes, in the case of the control EVLP. For SARS-CoV-2 infection in EVLP (3 donor lungs, donors 1, 2, 3), the right lung was connected to the circuit that was filled with 1.5 L of Steen solution supplemented with 1 g methylprednisolone, 1.5 g cefuroxime, and 7500 UI heparin. A flow rate at 24% of the theoretical cardiac output was applied at normothermia. The lung was ventilated at 4 ml/kg of donor body weight with a standard ICU-type ventilator equipped with a connected nebulizer with vibrating meshes (Aerogen^®^), for the delivery of SARS-CoV-2 (see “Viruses” section). The Steen perfusate (100 ml) was replaced with fresh one every 2 h. The left lung was placed at 4 °C and used for sampling before EVLP, designated as 0 h (see samplingsection). For control EVLP experiments (2 donor lungs, donors 4 and 5), the whole lung was connected to the circuit that was filled with 1.5 L of Steen solution supplemented with 1 g methylprednisolone, 1.5 g cefuroxime, and 7500 UI heparin. A flow rate at 40% of the theoretical cardiac output was applied at normothermia. The Steen perfusate (100 ml) was replaced with fresh one every 2 h. Views of the general experimental set up are shown in Additional file 2. Experimental set-up. This part of the study has been conducted between September 2020 and May 2021. Donors 1, 2, 3 were negative for SARS-CoV-2 virus, not vaccinated and without history of SARS-CoV-2 infection. Anti-viral IgGs were undetectable in their broncho-alveolar lavages (Additional file 3. ELISAIgG). The lungs from donors 4 and 5 were included in a previous study of our group [32].

The lung isolated monocyte/macrophage part of the study (Fig. 1B) was declared as a “dossier de Conservation et de préparation à des fins scientifiques D’Eléments du Corps Humain”: (CODECOH) DC N° DC-2020-3981 (1). Experiments on human tissues were approved by the regional investigational review board (Comité de Protection des Personnes Île de France VIII, Boulogne-Billancourt, France). Lung tissue samples were obtained from seven patients undergoing surgical resection for lung carcinoma at the Foch Hospital in Suresnes (France) (Additional file 1. Anamnesis). In line with the French legislation on clinical research and as approved by the investigational review board, all the patients gave their informed consent for the use of resected lung tissue for research.

### Viruses

The SARS-CoV-2 BetaCoV/France/IDF0372/2020 strain (passage 2 on Vero E6 cells), clade 19A, was obtained from the Pasteur Institute Paris, and has been isolated from a patient back from Hubei (China) at the Bichat hospital. The whole sequence is available under the accession number EPI_ISL_410720 (GISAID ID) and belongs to the original Wuhan lineage [33], that will be designated as WL (for Wuhan Lineage) in this paper. For the nebulization of donor 1’s lung (Fig. 1A), the virus stock (passage 4) was produced by amplifications in Vero E6 cells at an initial multiplicity of infection of 0.03 for 3–4 days at 33 °C in DMEM, centrifuged at 4000 g for 10 min 4 °C, aliquoted and stored at − 80 °C before titration (titer 2 × 10^7^ PFU/ml). For infection of monocytes/macrophages with WL (Fig. 1B), a second WL virus stock was similarly produced (titer 10^6^ PFU/ml). The SARS-CoV-2 viral strains UCN1 and UCN15 were isolated in March 2020, during the course of the active epidemic, from nasopharyngeal flocked swabs obtained at the University Hospital of Caen, Normandy, France as described in [34]. The spike nucleotide sequences of the UCN1 and UCN15 strains as well as of the WL sequence are provided in Additional file 4. Viral sequences show D614G mutations in both UCN1 and UCN15 strains. UCN1 and UCN15 will be designated as D614G-a and D614G-b in the rest of the paper. For the nebulization of D614G-a in donor 2, a passage 1 was used (titer 5.5 × 10^6^ PFU/ml) and for infection of monocytes/macrophages, a passage 2 was used (titer 10^6^ PFU/ml). D614G-b was only used for nebulization (donor 3) and was produced by polyethylene glycol concentration of a passage 2 production (titer 10^8^ PFU/ml), following a published protocol [35].

### Viral titration

For determination of tissue culture infective dose 50 (TCID50)/ml, Vero E6 cells (2 × 10^4^/well in 96-well plates) were inoculated with tenfold serial dilutions of viral suspension, cultured at 33 °C under 5% CO_2_ and after 96 h, the cells were fixed with 3.7% paraformaldehyde and stained with 0.2% crystal violet. The cytopathic effect was evaluated and the viral titers were expressed as 50% tissue culture infective dose per ml (TCID50/ml). For determination of particle forming units (PFU)/ml in a plaque assay, Vero E6 cells (3 × 10^5^/well in 12-well plates) were inoculated with tenfold serial dilutions of viral supernatants for 1 h, overlaid with 1.2% cellulose microcrystalline (Avicel, FMC BioPolymer) in Minimum Essential Medium Eagle + 2% FCS, cultured at 33 °C under 5% CO_2_ for 5 days. Following fixation and crystal violet staining, the number of plaques were counted, and the numbers of PFUs/ml were calculated by taking into account the dilution factor.

### Nebulization

Viral nebulization was done using a nebulizer with vibrating meshes, see the “Study design” section. The SARS-CoV-2 preparations were adjusted to 6.5 ml in DMEM and the total PFU amounts delivered were 1.2 × 10^8^ PFUs for WL (donor 1), 3.3 × 10^7^ PFUs for D614G-a (donor 2) and 10^7^ PFUs for D614G-b (donor 3) that were nebulized in the lungs for about 20 min. The potential effect of nebulization on viral infectivity was evaluated as follows: 10^6^ PFUs of WL were diluted in 6.5 ml DMEM and used for a control nebulization delivered to a 50 ml tube instead of to a lung. Three viral suspension samples were harvested before and after 30 min nebulization for viral detection using viral titration (TCID50) and RT-qPCR.

### Sampling during the EVLP part

Lung biopsies (2 g each) were taken from similar lung zones from the upper lobes (wedges) before EVLP (0 h), and after 10 h EVLP, cut in 4 pieces, placed in 10 ml HypoThermosol^®^ (STEMCELL Technologies, Vancouver, Canada) and kept on ice for 10 to max 24 h. This process was shown to maintain lung tissue stability up to 72 h for scRNA-seq [36]. In addition, 3 small biopsies (100 mg each) were placed in RNAlater at 0 h, 30 min, 5 h and 10 h after the end of nebulization, for RNA extractions. Perfusion liquid was collected every hour during EVLP and stored at − 80 °C. A broncho-alveolar lavage (BAL) was done with 100 ml PBS without Ca^2+ ^and Mg^2+ ^in the left lung (un-infected control). The aspirated BAL was spun for 10 min at 470 g at 4 °C and the supernatant was frozen at − 80 °C.

### Anti-SARS-CoV2 IgG detection

The BALs were thawed, spun at 10,000 g for 5 min, diluted 1:4 and assayed in duplicates using the SARS-CoV-2 Spike Protein IgG ELISA Kit from Elabscience (Houston, USA) as recommended.

### Lung cell isolation

For the EVLP part of the study, the lung tissue from biopsies kept in HypoThermosol was minced finely with scissors, placed in Multi Tissue Dissociation Kit 1 solution as recommended by the manufacturer (Miltenyi Biotec, Bergisch Gladbach, Germany), and incubated at 37 °C for 45 min in gentle agitation. The minced preparation was crushed on nylon mesh (1 mm) and filtered through successive nylon filters (500 µm, 100 µm, 40 µm). The cell suspension (referred as total cells) was washed in PBS (470 g, 15 min), processed to erythrocyte lysis, resuspended in RPMI + 2% FCS, filtered twice on 40 µm and counted by 3 independent measurements with a counting chamber. For donors 2 and 3, both at 0 h and 10 h, MHC class 2 positive cells (referred as MHC class 2^pos^) were enriched using the anti-human HLA-DR bead kit from Miltenyi Biotech (ref 130-046-101) and MS separation columns (ref 130-042-201), following the manufacturer recommendations. Briefly, 10 × 10^6^ purified lung cells were incubated with 20 µl beads + 80 µl PBS-EDTA-BSA buffer for 15 min, filtered on 40 µm, and loaded on MS column for positive selection. The total cells and HLA-DR-positively-selected cells were counted using a counting chamber and checked for viability using trypan blue, and showed over 90% viability.

For the isolated monocyte/macrophage part of the study, lung tissue was obtained from lobectomy at distance from tumors with the minimal possible duration of ischemia in the operating room. The lung tissue was immediately processed in the laboratory. Six grams of lung were minced and incubated for 45 min at 37 °C on a rotary shaker in RPMI 1640 supplemented with 100 IU/ml penicillin, 100 μg/ml streptomycin, 2 mM l-glutamine and 10% FCS containing 3 mg/ml collagenase D, 0.25 mg/ml Dnase I (Sigma-Aldrich) and 0.7 mg/ml dispase II (Gibco^®^, ThermoFisher Scientific, St Aubin, France). The minced preparation was crushed and filtered on a nylon mesh (1 mm diameter) and filtered through successive cell strainers (500 µm, 100 µm, 40 µm). Red blood cells were lysed with erythrocytes lysis buffer. After a wash in PBS, about 10^8^ cells (all instances over 90% viability) were kept in 10% FCS overnight at 4 °C before being used for staining, analysis or sorting.

### Monocyte/macrophage cell sorting and culture with SARS-CoV-2

Isolated human lung cells were stained with fluorescently labelled mAbs following a 15 min incubation at 4 °C with Fc block (1:4 ratio, Miltenyi Biotech). We used a mAb combination that was previously documented to identify lung monocytes and macrophage subsets [37]. The mAbs were diluted in RPMI containing 5% horse serum and 1% Hepes to a final dilution or concentration recommended by the manufacturers: anti-human CD45-FITC (clone HI30, 1/20), anti-murine CD11b-APC/Cy7 (clone M1/70, 5 µg/ml), anti-human CD206-APC (clone 19.2, 1/5), anti-human CD14-PE (clone TUK4, 5 µg/ml), anti-human CD16-Alexa700 (clone 3G8, 5 µg/ml), anti-human CD163-PerCp/Cy5.5 (clone GHI/61, 1/20), anti-human CD169-BV605 (clone 7-239, 1/20), anti-human CD43-PerCp/Cy5.5 (1G10, 1/20, BD Bioscience). For each mAb, a labelled isotype-matched control was used and the specificity of the labeling was controlled using the fluorescence minus one method. Dead cells were excluded by DAPI staining. The cell staining results were analyzed using FlowJo 10.8.1 software. The cell subsets were sorted from an initial number of 50–120 × 10^6^ cells using the “purity 1–2” mode of the CytoFLEX SRT sorter (Beckman Coulter). After centrifugation, the sorted cells were resuspended in X-VIVO 15 serum-free medium (Lonza), 100 U/ml penicillin and 1 µg/ml streptomycin and 5 × 10^4^ cells were plated per well in a 96-well plate. Cells were incubated at 33 °C, 5% CO_2_, with WL and D614G SARS-CoV-2 viruses at 0.1 and 0.001 MOIs, in duplicates, for 24 h (and for 48 h for viral detection). MOI 0.1 corresponds to a classical dose used in the literature, and MOI 0.001 corresponds to an estimate of the viral exposure per cell used in the EVLP part of the study (about 10^8^ PFUs for 10^11^ cells in the human lung [38]). In parallel the virus inoculum was checked for infectivity on Vero E6 cells. Before and at the end of the incubation, the cells were observed on a ZOE^™^ fluorescent imager (BIO-RAD). The culture supernatants were collected at 2 h, and 24 h (48 h in some cases) and stored at − 80 °C. Additional experiments were conducted in order to analyze IFN gene expression in the four monocyte/macrophage subsets. In that case, 10^5^ cells were plated per well in a 96-well plate in X-VIVO 15 serum-free medium (Lonza), 100 U/ml penicillin and 1 µg/ml streptomycin and incubated at 33 °C, 5% CO_2_, with medium alone or with D614G SARS-CoV-2 at 0.1 MOI or 0.001 MOIs, for 10 h.

### Cytokine detection

The supernatants of monocyte/macrophage cultures were assessed for detection of CCL2, CCL3, CCL4, CXCL8, CXCL10, TNFα, IL-1β, IL-1RA, IL-6, IL-10, IL-18 and IFN-α with a Human ProcartaPlex^™^ Mix&Match 12-plex (ThermoFischer Scientific, Waltham, MA) using a MagPix instrument (Luminex, Austin, TX) and the data were analyzed with the Bio-Plex Manager software (Bio-Rad, Hercules, CA). The detection limit for each cytokine was established from the lowest consistent calculated data by the BioPlex Manager software.

### Cell death analysis

After collecting the culture supernatant, the monocyte/macrophage cultures were incubated at 33 °C for 15 min with 200 µl of 2 µg/ml Sytox Green Nucleic Acid Stain (Invitrogen) diluted in HBSS. Images of wells incubated with Sytox or control solution were captured with a ZOE fluorescent imager and analyzed with the Image J software for determining the percentage of dead cells that stained with Sytox.

### Viral detection using RT-qPCR

SARS-CoV-2 RNA was detected using RT-qPCR from lung tissue or putatively infected suspensions (perfusion and nebulization liquids). Lung biopsies in RNAlater (100 mg) were placed in Trizol, homogenized with 1.4 mm ceramic beads in a Precellys 24 bead grinder homogenizer (Bertin Technologies), and purified using the NucleoSpin RNA kit that includes a DNAse digestion step (Macherey–Nagel, ref 740955.250). In the case of infected solutions, viral RNA was extracted from 100 µL using the NucleoSpin^®^ RNA Virus kit from Macherey–Nagel kit (ref 740956.250). The RT-qPCR for the E gene detection was done on 500 ng RNA from lung tissue in 25 µl final reaction volume and on 2 µl eluate of viral RNA from solutions in 10 µl final reaction volume, using the Superscript^™^ III Platinum^®^ One-Step qRT-PCR from in vitrogen (ref 11732-088) with forward primer E_Sarbeco-F1 ACAGGTACGTTAATAGTTAATAGCGT**,** reverse primer E_Sarbeco-R2 ATATTGCAGCAGTACGCACACA and fluorescent probe E_Sarbeco-P1 FAM-ACACTAGCCATCCTTACTGCGCTTCG-Tamra (Sigma-Aldrich, Merck) [39]. In the case of viral RNA detection from lung tissue, a SARS-CoV-2 RNA calibration curve of a previously titrated WL virus preparation was made with tenfold dilutions from 2000 to 0.2 PFU per reaction and PFU equivalent per 100 mg were calculated. Negative controls included control lung tissue RNA and H_2_O. In the case of viral RNA detection from suspension, a SARS-CoV-2 RNA calibration curve of a previously titrated WL virus preparation was made with tenfold dilutions from 500 to 0.05 TCID50 equivalent per reaction and TCID50 equivalent per ml were calculated. Negative controls included elution from the RNA extraction performed on control solutions and H_2_O. The cycling involved the following steps: reverse transcription at 55 °C for 20 min, denaturation at 95 °C for 3 min, amplification 50 cycles at 95 °C for 15 s and 58 °C for 30 s. A Cq value of 40 was attributed in cases of absence of detection (NA from the machine). The reactions were carried out in a CFX ConnectTM light cycler (BIO-RAD).

### IFN gene expression detection using RT-qPCR

Total mRNA from the SARS-CoV-2 exposed monocyte/macrophage subsets were extracted using the Arcturus (PicoPure^™^ RNA kit-ThermoFisher Scientific) and quantified by Qubit^™^ RNA high sensitivity kit (Invitrogen^™^, Fisher Scientific SAS, Illkirch, France). RNA (32 ng) was reverse-transcribed using random primers and the Multiscribe reverse transcriptase (Applied Biosystem, ThermoFisher Scientific). Quantitative real-time PCR was carried out on 1:4 of the RT reaction with 300 nM primers in a final reaction volume of 25 µl of 1 X SYBR Green PCR Master Mix (Applied Biosystem, ThermoFisher Scientific). We used the KiCqStart^™^ predesigned primer pairs H_IFNA1_1, H_IFNB1_1, IFNG, H_RPS18_1 from Sigma-Aldrich, Merck, Darmstadt, Germany. PCR cycling conditions were 95 °C for 30 s, linked to 40 cycles of 95 °C for 5 s and 60 °C for 30 s. Real-time qPCR data were collected by the Bio-Rad CFX Maestro system (Bio-Rad Laboratories Inc, Marne-la-Coquette, France) and expression of the different genes relatively to RPS18 and normalized to an internal calibrator (arbitrary units) were calculated by the 2^−∆∆Ct^ method.

### scRNA-seq and preprocessing of sequencing data

Single-cell suspensions were generated from 14 samples of the EVLP part of the study Additional file 5. Filtration results. For each sample, 2 × 10^4^ cells were loaded onto the 10X  Chromium to produce sequencing libraries, which were processed according to methods provided by 10X  Genomics (v3 Chemistry). Cell cDNA was sequenced using the Truseq Illumina Stranded protocol and the Illumina NextSeq 550 sequencing machine (> 3 × 10^8^ reads/sample). The reads were aligned with Cell Ranger v3.1.0 on the human genome using the GRCh38 assembly and the GTF file downloaded from Ensembl release 101, and on the genome sequence of the virus used for each infection. The 14 samples’ sequencing results were pre-processed and normalized using Seurat v4.3.0. Cells expressing less than 1000 genes were removed. Dead or lysed cells were excluded by removing cells with a percentage of mitochondrial genes above a threshold calculated using the Scater package (median percentage of mitochondrial genes across all individual cells + 3 median absolute deviations). Filtration of the data also included the removal of doublets using Scrublet (https://github.com/AllonKleinLab/scrublet [40], expected doublet rate set to 0.08), see Additional file 5. Filtration results for the results of the filtering procedure. The scRNA-seq raw data of donors 4 and 5 were included in a previous study of our group [32], however they were reprocessed together with the other data sets for the clustering strategies and definition of cell identities in the subsequent steps.

### Clustering strategies

We followed a similar workflow as the one used in our previous work [32]. Specifically, in order to correct for the donor and time effect, we integrated the 14 scRNA-seq samples (Additional file 5. Filtration results) using the FindIntegrationAnchors and IntegrateData functions in Seurat and we produced an “initial integrated UMAP” with 86,253 cells (Additional file 6. Cell identity determination). Parameters of the dimensionality reduction and graph-based clustering were adjusted (k.param = 12, resolution = 0.6) to obtain 22 clusters. Note that the anchor integration procedure was used only for integration of the 14 samples and assignment of cells to clusters. The whole dataset (86,253 cells) was subsetted in sub-objects per donor (5 sub-objects, donor 1 to 5). All downstream analyses used the expression values normalized for each donor separately, in order to avoid using the data transformed upon integration.

### Definition of cell identities in the scRNA-seq data sets

The original (cell × gene) matrices were pre-processed and normalized separately for each donor as described above and cells were assigned the cluster number of the “initial integrated UMAP”. Each donor data set was then analyzed with Azimuth, an automated reference-based algorithm for single-cell annotation (https://azimuth.hubmapconsortium.org/, version 2.0.0), using the Human Lung Cell Atlas as a core consensus reference model which encompasses 584,884 human cells of the lung and nose. The finest level of annotation was used. Cells presenting Azimuth annotation scores below 0.6 were discarded. We proceeded to a grouping of “close cell subtypes” in the cases of the B cells (B cells, plasma cells), stromal cells (smooth muscle, adventitial, peri-bronchial and alveolar fibroblasts), alveolar macrophages (alveolar macrophages, alveolar macrophage CCL3 + , alveolar macrophage MT-positive, alveolar macrophage proliferating), blood endothelial cells (arterial, aerocyte capillary, general capillary, venous systemic, venous pulmonary), and lymphatic endothelial cells (lymphatic differentiating, lymphatic mature, lymphatic proliferating). Each cell was associated with both a cluster number and a cell identity. In order to generate robust downstream analyses related to SARS-CoV-2 exposure, the cell identities representing less than 10% of a cluster in donors 1, 2 and 3 (EVLP with virus) and cells belonging to an identity/cluster not shared between donors 1, 2 and 3, were discarded for the subsequent analyses (Additional file 6. Cell identity determination). The cells selected with this Azimuth annotation-based process (66,737 cells) were projected onto the “integrated UMAP-filtered”, illustrated in Fig. 3A. Cluster C20 is absent in this “integrated UMAP-filtered” compared to in the “initial integrated UMAP” because C20 was discarded due to the heterogeneous cell identities found in this cluster. The contribution of each donor to the “integrated UMAP-filtered” is shown in Additional file 7. Representation of donors. The Azimuth-based cell identities (17 in total) that were kept for downstream analyses are: AMs, MoMacs, cMos, ncMos, DC2, mast cells, stromal cells, CD4^+^ T cells, CD8^+^ T cells, NK cells, B cells, blood endothelial cells, AT1s, AT2s, Transitional Club/AT2s, Club (non-nasal), Ciliated (non-nasal). As several Azimuth-based identities were found in several clusters, we considered the final identities on the basis of Azimuth identity and cluster belonging, leading to 28 identities. The representation of the 28 identities in each donor and timing is provided in Additional file 8. Identities per donor per timing. The top markers of the 28 analyzed cell identities found in the “integrated UMAP-filtered” were extracted separately for each donor, using the normalized expression values of each dataset before integration (top marker genes per cell identity versus the other identities (minimal log2FC ≥ 0.25, Bonferroni adjusted p-value ≤ 0.05)). The intersection of the top marker lists was then computed for each cell identity, to keep only common markers to all donors, ranked in a decreasing order using the lowest gene expression ratio among the donors (Additional file 9. Top expressed markers). An interactive viewer for visualizing the cells from the different donors, their cluster belonging, their identities and gene expression is available at https://applisweb.vim.inrae.fr/ICARE/.

### Statistics

The cytokine data were analyzed with R and were Log10-transformed. A Shapiro test was used to evaluate the normality of the data distribution in each group and timing. When the data did not pass the normality test, a non-parametric paired Wilcoxon test was used to compare the data between 2 groups. Alternatively, a paired t-test was used upon equal variance evaluation. The statistics of the genomic data are reported in the dedicated paragraph. The Fisher’s exact test was used to calculate the probability of finding viral reads in AMs and/or MoMacs among the cells from infected lungs.

## Results

### Establishment of the human ex vivo lung perfusion technique to study SARS-CoV-2 initial exposure in whole lung

In order to study the initial steps of SARS-CoV-2 infection in the human lung, taking into account the micro and macro-anatomical architecture and cell context, we implemented a technique used in lung transplantation, i.e. the ex vivo lung perfusion (EVLP) which maintains the whole organ alive and leads to fully satisfactory outcome results after lung transplantation [41]. As oedema may occur beyond 10 h of ex vivo perfusion and thus may compromise the lung quality [42], we used a 10 h duration of EVLP in the study (Fig. 1A).

**Fig. 2:**
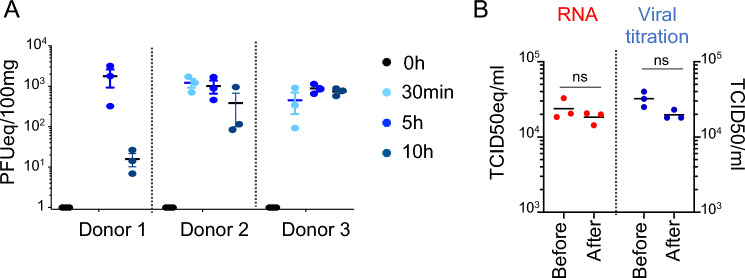
Analysis of SARS-CoV2 viral exposure upon infection of whole lung maintained alive and functional by ex vivo perfusion and ventilation. **A** Detection of SARS-CoV-2 genome in different lung biopsies after nebulization. From donor 1, 2 and 3 lungs, independent lung biopsies (100 mg each, 3 per timing) were collected in RNAlater before nebulization (0 h) and 30 min, 5 h and 10 h after the end of the nebulization (20 min duration). The 30 min time point was not done in case of donor 1. SARS-CoV-2 E gene was detected in the tissue RNA using RT-qPCR run in parallel to a calibration curve established from a titrated WL viral preparation, and the results were expressed in PFU equivalents (PFUeq) reported to 100 mg of lung tissue. **B** Control of viral infectious potential upon nebulization. A WL viral preparation (10^6^ PFU) was nebulized in 6.5 ml RPMI for 20 min in a collection tube in place of a lung. Three viral suspension samples were collected before the nebulization (Before) and 30 min after (After) the end of nebulization, viral RNA was extracted, subjected to RT-qPCR detection as described in A except that the results were expressed in TCID50eq/ml. In parallel, three other viral suspension samples (before and after) were titrated for their infectivity on Vero E6 cells and the results were expressed in TCID50/ml

Three human lungs declined for transplantation (Additional file 1. Anamnesis) were processed to EVLP as described in the Methods and at the onset of EVLP, each lung was infected with SARS-CoV-2 using a nebulizer (Additional file 2. Experimental set-up). All three donors were SARS-CoV-2-negative and anti-viral IgGs were undetectable in their broncho-alveolar lavages (Additional file 3. ELISAIgG). The 3 donor lungs received a different viral preparation, i.e. a Wuhan lineage (WL) and two D614G isolates (D614G-a and D614G-b) that had been minimally passaged in vitro (passage 4, 1 and 2 respectively) for limiting viral drift as much as possible (Additional file 4. Viral sequences). The 3 lungs received the maximal available doses that we could produce, i.e. 1.2 × 10^8^ PFUs for WL (donor 1), 3.3 × 10^7^ PFUs for D614G-a (donor 2), and 10^7^ PFUs for D614G-b (donor 3, see Discussion). In addition, 2 other lungs were used as control EVLPs as reported in [32] (donor 4, 5). The 5 lungs used in the study all displayed good macroscopical quality and respiratory function during the whole duration of EVLP (PaO_2_/FiO_2_ > 400 mm Hg). It is important to note that good quality lungs declined for transplantation are rare material for research, explaining the limited number of lungs in the study. SARS-CoV-2 RNA was detected by qRT-PCR in different samples of the lung wedges at 30 min, 5 and 10 h post nebulization, indicating a good dispersion of the inoculum (Fig. 2A). However, we were not able to detect active viral transcription or replication using this technique, as the level of viral RNA did not progressively increase during the 10 h period, but rather stabilized or decreased after reaching an initial peak. In addition, we checked that the nebulization preserved viral infectivity: indeed, no significant difference in plaque assay results was found between the viral suspension tested before and after nebulization (Fig. 2B). Finally, we could not detect viral RNA in the perfusion liquid even after 10 h, indicating the alveolo-capillary barrier was not permeable to the virus in our conditions (Additional file 10. PerfusionLiquid). Altogether exposure to SARS-CoV-2 of whole human lung can be achieved using the EVLP system during 10 h with stable maintenance of lung respiratory function.

### scRNA-seq composition of whole human lungs infected ex vivo with SARS-CoV-2 and controls

**Fig. 3:**
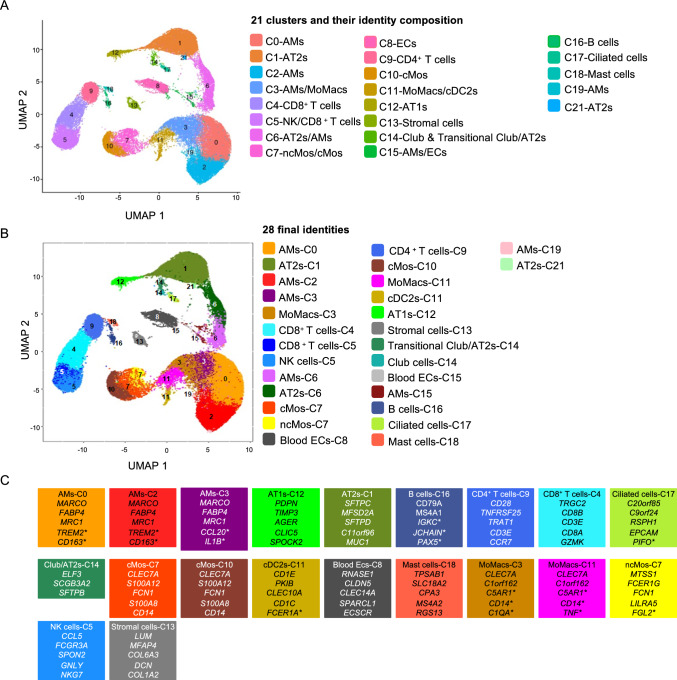
Single cell RNA-seq analysis of lung samples undergoing EVLP upon SARS-CoV-2 nebulization and control conditions, and definition of cell identities. **A** Cells were isolated from 5 donor lungs subjected to EVLP, at 0 h (no EVLP) and at 10 h EVLP; 3 lungs were nebulized at the onset of EVLP with SARS-CoV-2 (donor 1 with WL, donor 2 with D614G-a, donor 3 with D614G-b) and 2 lung EVLPs were conducted without virus (control EVLP for donors 4 and 5), see Fig. 1, and Methods. Donor lung samples (14 samples total) were used for 10X genomics scRNA-seq and processed for high quality transcriptomes, integrated, clustered and submitted to cell annotation analysis with the Azimuth package. A grouping of “close cell subtypes" identified by Azimuth was done (see Methods). Cells with low annotation scores (< 0.6), under represented identities (< 10% per cluster in donor 1, 2 or 3), and identities in clusters not shared between donors 1, 2 and 3 were excluded (see Additional file 6. Cell identity determination). The filtered cells were projected onto the "integrated UMAP-filtered" shown in A, with 21 clusters (C0 to C21, with C20 removed due to mix/undefined cell composition). For each cluster, the cell identity composition assigned by Azimuth is indicated. **B** The final cell identities defined based on Azimuth and cluster belonging are projected on the UMAP. A total of 28 final identities were obtained. **C** Top markers expressed by the major final identities. The italic gene names are shared with the canonical markers of the similar identities of two resource papers [43, 44]. Top markers with a * are representative of a cell type or of a cell type function/activation state reported by others: TREM2 and CD163 [45], CCL20 and IL1B [12], IGKC, J chain and PAX5 [46], PIFO [47], FCER1A [48], C5AR1, CD14, C1QA and TNF [14], FGL2 [32]

For studying the interaction of SARS-CoV-2 with lung at the resolution of single cells, we proceeded to scRNA-seq from biopsies of the lungs undergoing EVLP for 10 h after viral exposure (donor 1, 2, 3) and in control conditions (donor 4, 5) (Fig. 1A). Lung biopsies from the 0 h time point of the same donor (no EVLP) were included to assess the influence of 10 h EVLP ± virus on the cell responses. All biopsies were taken from similar upper lung zones and processed to single cell isolation; in addition, in donors 2 and 3, we added samples enriched in HLA-DR^pos^ cells at 0 and 10 h, for potentially increasing the number of virus positive-cells. We therefore obtained a total of 14 samples, which encompass the 0 h and 10 h total lung cell samples from the 5 donors as well as the 0 h and 10 h HLA-DR^pos^ cell samples from donors 2 and 3, see Additional file 5. Filtration results). The scRNA-seq were conducted on 2 × 10^4^ loaded cells from each sample using the 10X Genomics 3’end RNA-seq V3 chemistry. High-quality transcriptomes from 86,253 cells were generated upon removal of cell doublets and of dying cells based on the high proportion of mitochondrial gene expression (Additional file 5. Filtration results). In order to identify clusters corresponding to the same cell types across donors and timings, we integrated our 14 samples using a batch correction algorithm, generating an “initial integrated UMAP” with 22 clusters (Additional file 6. Cell identity determination). In order to determine the cell identity, we applied Azimuth to map our scRNA-seq data onto the Human Lung Cell Atlas (Human lung reference v2). Upon filtration of the data based on Azimuth scores and exclusion of minor identities (see Additional file 6. Cell identity determination and Methods), we generated an “integrated UMAP-filtered” (Fig. 3A) with 21 clusters (as C20 from the “initial integrated UMAP” was filtered out), and 17 cell identities (66,737 cells). All donors are similarly represented in the integrated UMAP-filtered (Additional file 7. Representation of donors). The clusters correspond to C0 AMs, C1 alveolar epithelial type 2 (AT2s), C3 AMs and MoMacs, C4 CD8^+^ T cells, C5 NK cells and CD8^+^ T cells, C6 AMs and AT2s, C7 ncMos and cMos, C8 blood and lymph endothelial cells (blood and lymph ECs), C9 CD4^+^ T cells, C10 cMos, C11 MoMacs and type 2 conventional dendritic cells (cDC2), C12 alveolar epithelial type 1 (AT1s), C13 stromal cells, C14 Club and Transitional/Club-AT2 epithelial cells, C15 AMs and ECs, C16 B cells, C17 ciliated epithelial cells, C18 Mast cells, C19 AMs, C21 AT2s. It is important to note that despite the use of Scrublet to remove doublets in the data processing (see Methods), C6 and C15 clusters included distant cell types. As the same identity assigned by Azimuth is found in different clusters and may correspond to cells in different activation/differentiation states, we defined the final cell identity based on the association of Azimuth identity and cluster belonging, leading to 28 final identities (Fig. 3B). In most cases, the 28 final identities are populated by cells from all donors at all timings, except in the case of very minor clusters (C19 and C21) and for Club cells in the control donor 5 (see Additional file 8. Identities per donor per timing). Notably, despite the merging of the data sets from HLA-DR^pos^ -enriched cells and total cells in the case of donor 2 and 3, the representation of cell identities is not different between the 5 donors (see Additional file 8. Identities per donor per timing).

Finally, the top expressed markers of the different final cell identities are provided in Additional file 9. Top markers. From these top expressed markers, a selection of hallmark genes of the cell identities is shown on Fig. 3C that was based on commonalities with (i) canonical markers defined in two resource papers from lung scRNA-seq [43, 44] and (ii) pertinent genes from other papers (see next). In particular AMs are associated with 4 clusters (C0, 2, 3, 6) indicating different activation states, that remain poorly defined as also reported by others [12]. The AMs-C6 signature is included in the AMs-C0 signature. C0, C2 and C3 express the AM canonical markers *MARCO, FABP4* and *MRC1* [43, 44]. The AMs-C0 and AMs-C2 express the *TREM2* and *CD163* genes that are markers of profibrotic AMs [45] and AM-C3 express inflammatory cytokine genes such as *CCL20* and *IL1B*, previously found to define a small pro-inflammatory AM subset in the airways of healthy human patients [12]. The MoMacs are associated with the C3 and C11 clusters and both share the expression of the canonical MoMac genes *CLEC7A* and *C1orf162* [43, 44] as well as the expression of *C5AR1* and *CD14* that are typical of macrophages derived from monocytes [14]. Notably C3 expresses complement genes such as *C1QA* and *C1QC* whereas C11  expresses inflammatory cytokine genes such as *TNF, CCL3, CCL20* (Fig. 3C and Additional file 9. Top markers). An interactive viewer for visualizing the cells from the different donors, their cluster belonging, their identities and gene expression is available at https://applisweb.vim.inrae.fr/ICARE/.

In conclusion, the analyzed human lungs in this study include the major cell types and subtypes of epithelial, myeloid, lymphoid, vascular and stromal cells that are expected in human lungs [43], with several identities such as AMs and MoMacs found in different clusters that appear to correspond to differences in cell activation states.

### SARS-CoV-2 RNA is associated with AMs and MoMacs in whole human lung infected ex vivo

**Fig. 4:**
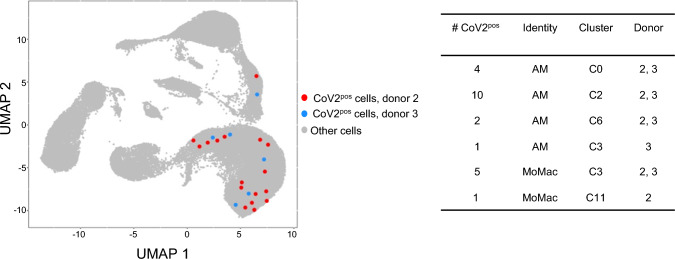
SARS-CoV-2 RNA is dominantly associated with macrophages upon whole lung infection. The SARS-CoV-2-positive cells (23 after filtration) were projected on the UMAP and the association with identities and clusters is shown. The viral sequences associated to the cells are reported in Additional file 11. ViralReadSeq

The SARS-CoV-2-positive cells were projected onto the “integrated UMAP-filtered” (Fig. 4). The 23 SARS-CoV-2-positive cells were found associated to AMs (C0, C2, C3, and C6) and MoMacs (C3 and C11), in the case of donors 2 and 3, with more virus-positive cells originating from donor 2 (17 for donor 2, 6 for donor 3). Few virus-positive cells (4) had been removed from the filtration based on Azimuth score, thus corresponding to cells of poorly defined identities that we did not consider.

No virus-positive cells were found in the case of donor 1 (WL strain). Statistical analysis using Fisher’s exact test showed that the false discovery rate for SARS-CoV-2 RNA associated with AMs or MoMacs among all cells from infected lungs was 2.58 × 10^–8^, with AMs-only 4.7 × 10^–4^, and with MoMacs-only 1.3 × 10^–2^. The viral sequences associated to the cells are reported in Additional file 11. ViralReadSeq. There was no effect of the HLA-DR^pos^ enrichment on viral association with cells. As viral association with pneumocytes was expected from other studies, we performed viral RNA detection on the total scRNA-seq data, i.e. before cell filtration based on mitochondrial gene expression and gene number cut-offs (Additional file 12. COV2pos_before_filtration). Doing so, 49 virus-positive cells were found, among which 38 were assigned as macrophages and 10 as possible pneumocytes. However, the gene number per pneumocyte was too low (< 100) to consider them as cells, they originated from a single sample, and the level of the score calculated by Azimuth was below confidence (< 0.5). Therefore, our data do not permit to formally identify pneumocytes as primary targets of the virus, but they do not exclude them (see Discussion). In any event, our data support that AMs and MoMacs are dominant primary targets of SARS-CoV-2 in whole infected lungs.

We retrieved the normalized gene expression of SARS-CoV-2 known receptors and sensors across the different lung identities for each donor, i.e. Sialic Acid Binding Ig Like Lectin 1 (SIGLEC1, CD169), Dendritic Cell-Specific ICAM-3-Grabbing Non-Integrin 1 (DC-SIGN, CD209), Asialoglycoprotein Receptor 1 (ASGPR1), ACE2, Toll-like Receptor (TLR)2 and TLR4, Basigin (BSG, CD147), Fc Gamma Receptor IIIa (FCGR3A), AXL Receptor Tyrosine Kinase (AXL), Neuropilin 1 (NRP1), Transmembrane Serine Protease 2 (TMPRSS2), and Transmembrane Protein 106B (TMEM106B) (Additional file 13. Viral receptors). The viral reads were found associated with cell identities (AMs and MoMacs) that globally express relatively higher levels of FCGR3A, BSG (CD147), NRP1, AXL, FCGR3A, BSG and TLR4, as compared to other cell identities (Additional file 13. Viral receptors). However, at the single cell level, cells with viral reads were not found systematically associated with high mRNA expression of SIGLEC1, FCGR3A, NRP1, AXL, BSG nor TLR4, within any of the cell type nor across all cell types. Donor 1, for whom no viral read could be detected, expressed similar levels of the putative SARS-CoV-2 receptors across cell identities as donors 2 and 3, therefore a lack of receptor expression at the mRNA level in this donor does not explain lack of SARS-CoV-2^pos^ cells (Additional file 13. Viral receptors).

Overall, SARS-CoV2 virus is found associated with AMs and MoMacs and not with epithelial cells in the context of whole lung infection at early time points, indicating a major tropism of this virus for these two myeloid cell types. However, the association with AMs and MoMacs at the single cell level was not systematically related to the relative high expression of a given putative receptor, at the mRNA level.

### Lung monocyte/macrophage subsets differentially respond to SARS-CoV-2, with lung MoMacs producing higher inflammatory cytokine levels than AMs

**Fig. 5:**
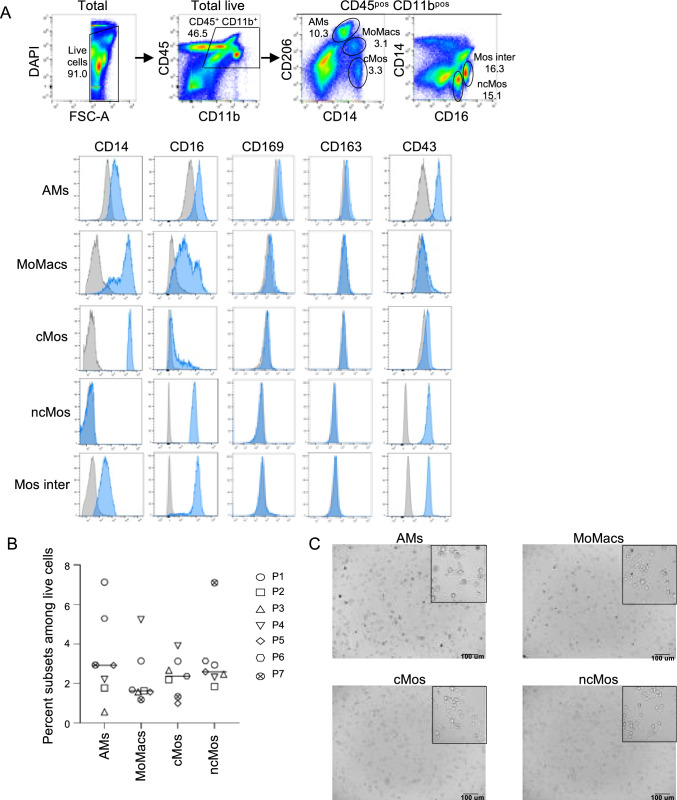
Lung monocyte/macrophage subset characterization and sorting. **A** Gating strategy and expression of markers on lung monocyte/macrophage subsets (representative patient). Lung cells were isolated from lung biopsy obtained upon lobectomy, and stained with a combination of the following conjugated mAbs: anti-CD45-FITC, anti-CD11b-APC/Cy7, anti-CD206-APC, anti-CD14-PE, anti-CD16-Alexa700, anti-CD163-PerCp/Cy5.5, anti-CD169-BV605, anti-CD43-PerCp/Cy5.5. For each mAb, a labelled isotype-matched control was used and the specificity of the labeling was controlled using the fluorescence minus one method. Dead cells were excluded by DAPI staining. From the live CD45^pos^CD11b^pos^ cell gate, AMs were identified as CD206^hi^CD14^lo^ cells, MoMacs as CD206^int^CD14^hi^ cells, cMos as CD206^neg^CD14^hi^ cells, ncMos as CD16^pos^CD14^neg^, and intermediate monocytes (Mos inter) as CD16^pos^CD14^pos^ cells. The staining intensity of the different subsets for CD14, CD16, CD169, CD163 and CD43 is shown in blue histograms overlaid on their respective isotype control histogram in grey. **B** The  percentage of AMs, MoMacs, cMos and ncMos is reported for 7 patients used for cell subset sorting. **C** Images of AMs, MoMacs, cMos and ncMos plated in 96-well plates were captured with a Zoe Cell Imager (× 20) and the areas marked with a black square correspond to higher magnification (× 60)

An analysis of the differentially expressed genes between the 3 virus-exposed and 2 control lungs after 10 h EVLP, as well as Gene Set Enrichment Analysis, did not consistently retrieve enriched biological pathways that would be specific to the viral exposure and not to the EVLP procedure. Several hypotheses can be proposed to explain this failure that will be discussed next. In order to analyze and compare the response of lung AMs and MoMacs to an initial exposure to SARS-CoV-2, we isolated and purified these subsets as well as cMos and ncMos, which are close cell types, from human lungs undergoing lobectomy (7 patients). The gating strategy for sorting these subsets has been established based on the one of the Ankit Bharat’s group in Chicago [37] and Patrick Hume’s group at NIH [10] and is reported in Fig. 5A. The expression of monocyte/macrophage markers on the subsets is shown on a representative example (see the legend of Fig. 5A for the gating strategy) and supports their identity: AMs express higher expression of CD169, CD163 and CD43 than the other subsets, MoMacs express higher levels of CD14 and lower levels of CD206 than AMs, cMos express the highest level of CD14, and ncMos display the highest levels of CD16. The intermediate Mos (CD16^pos^CD14^pos^), whose function remains poorly understood, were not considered in the next steps, because the CytoFLEX SRT can only sort 4 populations. In 7 patients, the proportion of the different sorted subsets among lung live cells laid between 0.6 and 7.1% for AMs (3.3 ± 2.2%, mean ± sd), 1.2 and 5.2% for MoMacs (2.3 ± 1.4%), 1 and 3.9% for cMos (2.4 ± 1%) and 1.8 and 7.1% for ncMos (3.2 ± 1.8%), Fig. 5B. Therefore, the different monocyte/macrophage subsets represented less than 8% of the lung live cells; however, their respective proportions varied between patients.

**Fig. 6:**
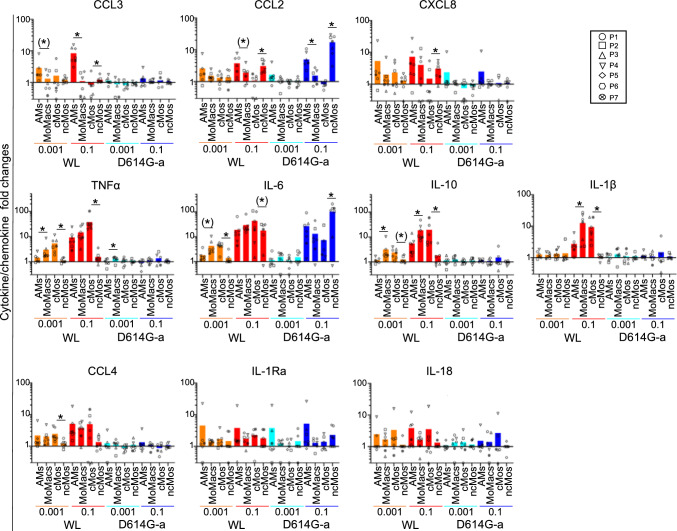
Cytokine/chemokine fold changes induced by SARS-CoV-2 in lung monocytes/macrophages, depending on viral doses, viral strains and cell subsets. The sorted lung AMs, MoMacs, cMos, ncMos (5 × 10^4^, duplicates) from 7 patients were exposed to SARS-CoV-2 WL and D614G-a at 2 MOI, i.e. 0.1 and 0.001. The supernatants from mock cultures and viral exposed cultures were collected after 24 h and subjected to cytokine detection using Human ProcartaPlexTM Mix&Match 12-plex. The detection limit for each cytokine was established from the lowest calculated data by the BioPlex Manager software. For the different cytokines/chemokines, for each subset in each condition, a ratio between the stimulated and mock culture was calculated, Additional file 16. Means ± sd. The ratios were log transformed and analyzed with R. A Shapiro test was used to evaluate the normality of the data distribution in each group and timing. When the data did not pass the normality test, a non-parametric paired Wilcoxon test was used to compare the data between 2 groups. Alternatively, a paired t-test was used upon equal variance evaluation. The statistical results of paired comparisons are reported in Additional file 17 and 18. The p-values between the AMs and MoMacs and between the cMos and ncMos are reported on the figure, as * when < 0.05 and as (*) when comprised between 0.08 and 0.05. The cytokine result panels were grouped as follows: top lane with several AM values > MoMac values and ncMos values > cMo values, mid lane with AM values < MoMac values and ncMos values < cMos values, bottom lane without clear pattern

The cells of the 4 different monocyte/macrophage subsets were placed in culture (5 × 10^5^ per 96-well, Fig. 5C) and exposed to SARS-CoV-2 for 24 h. Two of the viral strains nebulized in the whole lung model were used, i.e. WL and D614G-a, at a conventional dose generally found in the literature (0.1 MOI) and at a dose similar to the one used in the nebulized lung (0.001 MOI, see Methods). After 24 h, no cell death was induced by the viruses and no production of viral particles could be detected in any of the subset, nor after 48 h (Additional file 14. Survival viral production). After 24 h, chemokine and cytokine productions were measured in the supernatants using the Luminex/Multiplex technology. IFNα and CXCL10 were not detected and thus not illustrated. In addition, no induction of *IFNA, IFNB* and *IFNG* gene expression could be detected in the 4 monocyte/macrophage subsets exposed to SARS-CoV-2 during 10 h (Additional file 15. IFN-qPCR). We first analyzed the response of each subset to WL and D614G-a according to the viral dose by calculating the fold change of cytokine/chemokine expression upon viral exposure over mock condition and we compared these fold changes between subsets, viral doses and viral strains (Fig. 6, see Additional files 16 for means ± sd and Additional files 17 and 18 for detailed p-values). For 3 chemokines, i.e. CXCL8, CCL3 and CCL2, MoMacs presented lower fold changes than AMs and cMos presented lower fold changes than ncMos in most instances (see Fig. 6, Additional files 16 and 17). Conversely for 4 cytokines, i.e. TNFα, IL-6, IL-10 and IL-1α, a reverse pattern was observed: MoMacs displayed higher fold changes than AMs and in most cases cMos displayed higher fold changes than ncMos (except in the case of IL-6 with D614G-a). No clear pattern was observed for CCL4, IL-1RA and IL-18. Overall, MoMacs and cMos behaved similarly. For all subsets across the analyzed chemokine/cytokines, higher fold change responses to WL than to D614G-a were obtained (in 80 comparisons throughout conditions, 35 showed higher responses to WL than to D614G-a versus only 3 in the reverse orientation, p-values < 0.079, see Additional file 18); only CCL2 and IL-18 did not follow this trend. Finally, higher responses were obtained with the 0.1 MOI dose for WL (20 out of 40 comparisons, p-values < 0.059 with only one in the reverse orientation), and less in the case of D614G-a (7 out of 40 comparisons, p-values < 0.067, with 2 in reverse orientation, see Additional file 18).

**Fig. 7:**
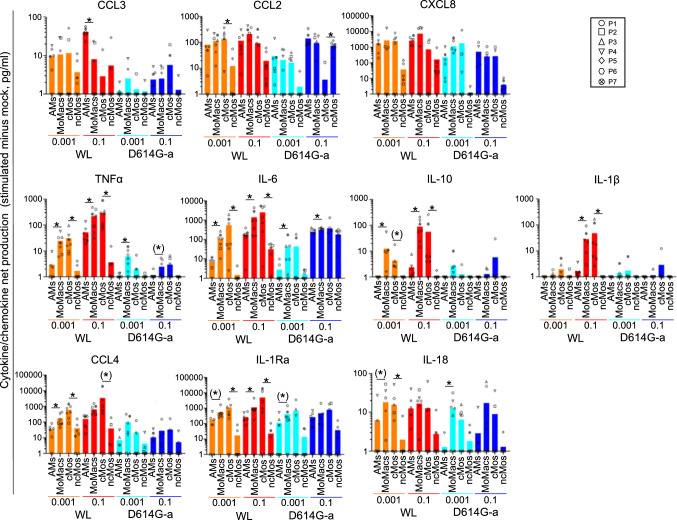
Cytokine/chemokine net production induced by SARS-CoV-2 in lung monocytes/macrophages, depending on viral doses, viral strains and cell subsets. For the different cytokines/chemokines analyzed with the human ProcartaPlexTM Mix&Match 12-plex, for each subset in each condition, the difference of cytokine levels between the stimulated and mock culture was calculated and analyzed as in Fig. 6, Additional file 16. Mean + sd. The statistical results of paired comparisons are reported in Additional file 19 and 20. The p-values between the AMs and MoMacs are reported on the figure, as * when < 0.05 and as (*) when comprised between 0.08 and 0.05. The cytokine result panels were grouped as follows: top lane with AM values > or not different to MoMac values, mid and bottom lanes with AM values < MoMac values

While the expression fold changes between virus-stimulated versus mock conditions inform on the response of the different subsets to the virus, the viral effect on the global production of the cytokine in the milieu is better reflected by differences of cytokine levels between stimulated and mock conditions (= net production, Fig. 7, see Additional files 16 for means ± sd, and Additional files 19 and 20 for detailed p-values). Indeed, for instance, as MoMacs produced higher net production of CCL4 and IL-1RA in mock conditions than AMs, the cytokine fold changes are not different between the 2 subsets, however the net production of these cytokines under stimulation of MoMacs is higher than the one of AMs (p-value < 0.072 in several cases). The analysis of net cytokine/chemokine productions shows that for the 3 chemokines CXCL8, CCL3 and CCL2, no clear/consistent differences between AMs and MoMacs nor between ncMos and cMos were found (Fig. 7, Additional files 16 and 19). Conversely, for 7 cytokines/chemokine, i.e. TNFα, IL-6, IL-10, IL-1α, CCL4, IL-1RA and IL-18, MoMacs presented higher net production values than AMs, MoMacs generally did not much differ from cMos, and ncMos presented lower net production values than all other subsets in most instances (Fig. 7, Additional files 16 and 19).

This mode of analysis also shows a higher net production in response to WL than to D614G-a (in 80 comparisons, 35 showed higher responses to WL than to D614G-a versus only 2 in the reverse orientation, p-values < 0.073, Additional file 20). Higher net production responses to viral stimulation were also obtained with the 0.1 MOI dose for WL (18 out of 40 comparisons, p-values < 0.062), and less in the case of D614G-a (9 out of 40 comparisons, p-values < 0.075).

Overall, the monocyte/macrophage subsets, especially the AMs and MoMacs, appeared differentially represented between patients and they all produced inflammatory chemokines and cytokines upon SARS-CoV-2 stimulation. MoMacs revealed to be the highest inflammatory molecule producers among the different lung monocyte/macrophage subsets. The monocyte/macrophage responses also depended on the viral strain and dose.

## Discussion

Our work provides novel insights into the initial stages of SARS-CoV-2 interaction with the human lung, the primary target organ for virus-induced pathology. We employed original approaches, including the infection of the whole lung and the infection of lung-derived monocyte/macrophage subsets. These methods have not yet been used for that purpose, to the best of our knowledge. Our results show that (i) AMs and MoMacs are major targets of SARS-CoV-2 in the initial step of lung infection, (ii) both AMs and MoMacs as well as cMos and ncMos produce inflammatory cytokines upon exposure to the virus and (iii) MoMacs are the highest producers and ncMos the lowest. These results indicate that both AMs and MoMacs can capture the nebulized virus from the lumen, consistent with previous observations for other antigens and pathogens [6, 7]. MoMacs, which appeared to be the most responsive subset in terms of inflammatory chemokines/cytokines upon SARS-CoV-2 exposure in this study, are variably represented among lung cells (Fig. 5), possibly reflecting the patients’ pathological history [5, 10, 49]. The relative abundance of MoMacs and the extent of the response of AMs and MoMacs may initiate a combination of cellular and molecular sequences leading to COVID-19 severity. Our results also indicate that, in addition to the monocyte/macrophage subset representation, the viral dose and viral strain can impact on the magnitude of the inflammatory response by lung macrophages.

Our study based on infection of whole human lung, while being attractive due to the preserved spatial relationships between virus and cell types, revealed to present several limitations in the current experimental settings. First, viral parameters can have affected the conclusions, such as the viral dose that we used for nebulization. We used the maximal dose of our viral productions that could be nebulized (10^7^ to 1.2 × 10^8^ PFUs), taking care of limiting the in vitro passage and amplification to avoid viral drift (passage 1 to 4). The viral dose received by the lung in patients is not known and it depends on a first replication in the upper respiratory tract [50]. In a study of viral inoculation in human volunteers [51], the virus expression initially rose in the nose, peaking at ~ 8.87 Log10 copies per milliliter. As viral copies reach at least 10 times higher values than PFUs/ml, the amount that we administered thus lays in a clinically relevant range. However, in our model, the virus was administered in a unique dose, whereas in real life, it can be expected that the virus produced in the upper respiratory tract repeatedly reaches the lung. Also, the viral strain and mode of production may affect the viral tropism. All our viruses were produced on Vero E6 cells, yet no viral read was found associated to lung cells in the case of infection with WL, despite the high dose used (1.2 × 10^8^ PFUs). It is possible that the D614G mutation in the spike, that was shown to be associated to a higher viral infectivity and transmissibility [52], may have favored the infectivity in our whole lung model with the D614G-a and -b viruses.

Another limitation of the whole lung infection model is the duration of EVLP that cannot be reliably prolonged beyond 10 h, as oedema may develop, compromising the functionality of lung areas. Although this duration is compatible with the rapid viral replication in culture [53], it may not be the case in a whole organ and this duration may thus not be sufficient to see virus-induced cell responses nor replication. Indeed, we could not find any increase in viral RNA between 30 min and 10 h post nebulization (Fig. 2). Furthermore, we realized that the EVLP per se induces a significant response of the different cell types during the 10 h procedure, as we recently published [32]. This spontaneous response to EVLP, likely related to ischemia–reperfusion, possibly interfered on the response to the virus. However, this caveat also applies to ex vivo cell cultures of lung tissue in general (explants, tissue slices) that unavoidably leads to hypoxia-reoxygenation stress responses, a feature that is usually overlooked.

The donor status has importance in the obtained results. We assessed the initial stages of viral infection with “naïve donor lungs”, in experiments performed in 2020–2021, when such statuses were still available. Indeed the lung donors were negative for SARS-CoV-2 ongoing infection, not vaccinated and without history of SARS-CoV-2 infection, and did not have anti-spike IgGs in their broncho-alveolar lavages (Additional file 3.IgGElisa). Therefore, the apparent lack of viral replication in our whole lung model cannot be explained by interference with adaptive immunity. In addition, we can conclude that the viral reads from our scRNA-seq data correspond to the nebulized virus and not to reactivation of a past infection [54–56]. However, it should be emphasized that the lungs from human donors refused for transplantation were by essence heterogeneous (Additional file 1. Anamnesis): they were from patients with different ages (41 to 84 years old), with probable different pathological/exposition history, and different duration in intensive care units. All these parameters may have had an impact on the lung cell responses to EVLP, with and without virus. It is thus conceivable that specific biological parameters of donor 1 may explain the lack of viral detection with scRNA-seq in the lung at 10 h. In the case of the patients undergoing lobectomies, their serological status was not tested. Therefore, the inflammatory responses of isolated monocyte/macrophage subsets of some of these patients may have been modified by a previous SARS-CoV-2 infection, that could have reprogramed lung macrophages to a trained status via epigenetic modifications [57].

Interpretation of the results of the whole lung model depended on the constraints of the scRNA-seq 10X  Genomics 3’ chemistry v3 technique. In each sample, a maximum of 20,000 cells can be analyzed that represent a small fraction of the total lung cells (10^11^, [38]). We detected few virus-positive cells in the scRNA-seq data, i.e. 23 virus-positive cells among the 66,737 cells of the “integrated UMAP-filtered”. A low proportion of virus-positive cells was also found in the scRNA-seq data of the lungs of SARS-CoV-2 infected ferrets between day 2 and 5, even lower than here [58]. However, as the human lung contains about 1.5 × 10^7^ times the number of analyzed cells with scRNA-seq, extrapolation by calculation leads to a quite high theorical number of virus-positive cells in the entire lung (> 30 × 10^7^). In addition, the data from scRNA-seq only capture a fraction of the transcriptome of each cell, a caveat designated as drop-outs [59, 60]. Therefore, the number of viral positive cells may have been under-estimated. Besides, while the scRNA-seq data of the HLA-DR^pos^ -enriched and of the total cells were merged in the case of donors 2 and 3, this merging did not result in a higher proportion of macrophages and had no significant impact on the cell identity distribution across donors (Additional file 8. Identities per donor per timing, see the relatively low proportion of macrophages in donor 3 at 0 h). Furthermore, viral reads in donors 2 and 3 were found in the cells that initially originated both from the total and the HLA-DR^pos^ -enriched cell samples (Additional file 11. ViralReadSeq). The lack of detectable effect of the HLA-DR^pos^ enrichment can be explained by (i) the expression of HLA-DR by many lung cell types including epithelial cells [61, 62], (ii) the lack of discrimination between high and low expressors by the immunobead selection that we used, (iii) the intrinsic variation of cell identity representation between donors.

The scRNA-seq method used here together with the limitation of the current knowledge on lung macrophages may not have taken well into account the complexity of the AMs and MoMacs activation states, nor their exact localization in the airway, alveola, parenchymal, peri or intra-vascular sites [5, 11, 43]. The clustering of the scRNA-seq indicates the presence of AMs and MoMacs in different activation/differentiation states, and the MoMacs in our datasets most probably correspond to IMs from mixed niches (bronchial, alveola, peri-endothelial, peri-nervous bundles etc.). Additionally, the C6-AMs and C6-AT2s were located in the same C6 cluster, away from the other AMs and AT2 clusters; while Azimuth assigned these C6 cells as distinct AMs and AT2s cell types, with confirmation by their top-gene expression (Additional file 9. Top markers), their location in the same cluster indicates some degree of transcriptomic proximity. These cells, that also encompass virus-positive cells, may correspond to cell doublets not removed by the Scrublet algorithm or to possible efferocytosis of epithelial cells by AMs.

While our results show that AMs and IMs are major initial targets in the preserved architecture of a whole lung, it is possible that other cell types such as pneumocytes were missed (Additional file 12. COV2pos_before_filtration). Indeed, the representation of cell types using scRNA-seq can be biased, due to the cell preparation process (enzymatic treatment) and the bio-informatic filtration (cut-off on mitochondrial genes’ representation, cut-off on the gene number per cell, Azimuth score threshold). Here the proportion of macrophages (39 ± 20%) is higher than that of epithelial cells (22 ± 15%), see Additional file 8. Identities per donor per timing, whereas conventional morphometric analyses indicate that the number of lung epithelial cells is about twice that of macrophages [38]. In addition, the viral capture by macrophages may be favored by the position of the AMs in the alveola and their expression of suitable receptors (see next). Therefore, the low proportion of epithelial cells in our data set and biological parameters decreased the chances to reliably detect the viral association with AT2s and/or AT1s in our system.

We could not retrieve consistently modified biological pathways induced by the virus in the different subsets, both using functional genomic analysis with differentially expressed genes and high-throughput gene set enrichment analysis. The aforementioned limitations probably collectively account for this failure, i.e. the potential inadequacy of the viral dose, the low proportion of virus-positive cells within macrophages precluding detection of modulated pathways in the whole subset, the restricted EVLP duration of 10 h, the intrinsic cell subset response to EVLP, the variations between donors arising from ICU conditions, and also the limited number of donors (3 virus-exposed lungs versus 2 control lungs). Future experiments using this whole lung infection should consider more donors (for control and infected lungs), higher viral doses, and spatial transcriptomic technics to better take into account the cell type location in the organ. It is also possible that more infectious viruses for the lung, such as influenza virus, would give different pictures of cell responses.

Our results highlight that AMs and MoMacs are primary targets of the virus and that they present distinct inflammatory responses. In the context of the current literature, these results align with previous reports but conflict with others. Indeed, several studies showed that exposure of monocytes/macrophages to SARS-CoV-2 triggered the synthesis of inflammatory cytokines, as we found [63–68], whereas others reported that it did not [69–73]. Most of the studies cited above implicated macrophages differentiated in vitro from monocytes, and may not display the same functional properties as monocytes and macrophages imprinted by the lung tissue. Furthermore, whereas the in vitro M1 and M2 polarized macrophage binary types do not embrace the in vivo complexity [14], the M2 macrophage type (generated with M-CSF) appears unable to respond to SARS-CoV-2, in contrast to the M1 macrophages (generated with GM-CSF) [70, 74, 75], highlighting the impact of the macrophage activation state/polarization on the response to SARS-CoV-2. Indeed, very recently, another paper reported the particularly elevated inflammatory transcriptomic program induced by the virus, specifically in interstitial lung macrophages [76]. Furthermore here, we showed that the cytokine/chemokine production levels induced by SARS-CoV-2 segregated into two groups, one group with similar levels produced by AMs and MoMacs (CCL2, CCL3, CXCL8), and the other group with higher levels by MoMacs than AMs (CCL4, IL-1α, IL-1RA, IL-6, IL-10, IL-18, TNFα). The panel of cytokine/chemokine response that we tested was limited to 12 cytokines based on previous knowledge [77], and we may have missed other selective types of responses by subsets. Notably we found that the different monocyte/macrophage responses in inflammatory cytokines were higher upon exposure to WL than to D614G virus, indicating that the response of lung monocyte/macrophage subsets also varies depending on viral strains. The pathogenicity of viral strains has decreased since the one of the original Wuhan type [78], and it might be related to a decreased propensity of the daughter viral strains to activate lung monocytes/macrophages. In any case, no IFN-α protein nor *IFNA*/B gene upregulation could be detected, confirming the results of others regarding the responses of macrophages to SARS-CoV-2 [79, 80]. Indeed, several SARS-CoV-2 proteins impede type I/III IFN induction through interfering on the interferon regulatory factor 3 and 7 pathways, whereas the activation of the nuclear factor-κB (NF-κB) pathway is maintained, as demonstrated by single-cell ATAC sequencing [81, 82]. It has been proposed that the NF-kB pathway, that drives the transcription of inflammatory cytokine genes, is required for the virus replication cycle [82].

The difference of responses between monocyte/macrophage subsets that we observed, and between studies that used different culture modalities, may pertain to differential expression of receptors, sensors and surface lectins. Several studies reported that some monocyte/macrophage types expressed Angiotensin Converting Enzyme 2 (ACE2) [29, 70], the most documented SARS-CoV-2 receptor, and that this expression conditioned the monocyte/macrophage response to the virus [30]. However, expression of ACE2 on monocyte/macrophage is debated [29, 30, 67, 70, 73, 83], and was very weak in our scRNA-seq analysis (Additional file 13. Viral receptors). Others documented that SIGLEC1 (CD169) on macrophages restricted the entry of the virus in these cells that express pro-inflammatory cytokines upon sensing via the MAVS-dependent pathway [68]. Capture of apoptotic bodies from infected cells was shown to be a determining mechanism in macrophage response to SARS-CoV-2, a property that also may depend on receptors for apoptotic bodies on macrophage types [71]. Toll-like receptors (TLRs) have been associated with the pathogenesis of COVID-19 [84] in particular TLR2 and 4 that are expressed by macrophages. Inflammation in the lung was shown to be mediated by TLR2, independently of SARS-CoV-2 replication, either through the binding of the E envelop protein [85] or of the S spike protein [86]. In addition, the S spike protein also binds TLR4 on human and murine macrophages and induces a high production of pro-inflammatory cytokines [87–90]. Recently viral peptide fragments making complexes with double stranded RNA were found to strongly promote TLR3 signaling and to grossly amplify inflammatory responses [91]. Finally, the viral ORF8 virokine triggers pro-inflammatory cytokine synthesis through MyD88, a major transducing molecule of the TLR pathway [92], and also directly binds the NLR family pyrin domain containing 3 (NLRP3) in human monocytic cells [93]. In the present study, viral association was not systematically found related to high expression, at the single cell level, of any of the putative viral receptors ACE2, TMPRSS2, CD209, TMEM106B, TLR2, ASGR1, SIGLEC 1, FCGR3A, BSG, NRP1, AXL, and TLR4 (Additional file 13. Viral receptors). However, the drop-out caveat of scRNA-seq mentioned above may have masked possible correlations. In addition, mRNA abundance may not reflect the level of protein expression.

We could not detect productive infection in the monocytes/macrophages isolated from donor lungs and exposed in vitro to the virus (Additional file 14. Survival & viral production); this is the most consensual finding among reports dealing with monocytes/macrophages despite exceptions [29, 94]. Several studies reported that SARS-CoV-2 developed an abortive cycle in macrophages, with accumulation of genomic and subgenomic RNA and viral proteins but no production of infectious particles [66–68]. The abortive cycle of SARS-CoV-2 in macrophage culture was sufficient to trigger inflammatory pathways via different documented sensing pathways [68, 85, 93]. Interestingly, SARS-COV-2 may propagate through filiform extensions between macrophages without being released [54]. Upon viral exposure, we could not find evidences for viral induction of cell death (Additional file 14. Survival & viral production), as reported by some authors [29] but at odds with others [63]. However, we obtained substantial spontaneous cell death after 24 h culture (about 40–50%). We cultured the cells in X-vivo 15 medium, a serum-free medium adapted to immune cells, without addition of growth factors. Addition of M-CSF could have improved the viability; however, M-CSF was shown to favor a M2 profile [95], that might interfere on SARS-CoV-2 responses [70, 74, 75]. While such a level of cell death is expected upon primary cell isolation, sorting and culture for 24 h, it may have affected the results, yet the level of cell death was similar between all monocyte/macrophage subsets (Additional file 14. Survival & viral production).

Based on our results, we propose a model in which the initial tropism of SARS-CoV-2 for lung AMs and MoMacs play a pivotal role in the infection’s outcome, leading to severe COVID-19 in some individuals. In that model, the interaction of SARS-CoV-2 with lung macrophages would lead to an initial burst of chemokine and cytokine synthesis; the intensity and nature of this initial response would depend on the relative abundance of MoMacs and the intrinsic response of AMs and MoMacs, both of which varying between individuals, shaped by genetic and environmental factors, and medical history. This first chemo/cytokinic response would lead to the recruitment of different types of leukocytes, setting the ground for the development of alveolitis [96]. The recruited leukocytes include monocyte subsets (CD14^pos^HLA-DR^low^cMos and CD16^pos^ ncMos), that were correlated with severity [19, 24, 66, 97]. In addition to cell recruitment, the initial production of IL-10 by MoMacs and AMs would enhance the expression of ACE2 on AMs, favoring second rounds of viral capture and responses in macrophages [21]. In parallel, the infection of AT2s, directly through binding to ACE2 or possibly through a trans-infection process mediated by SIGLEC1 expressed by macrophages [98], would produce viral progenies in the context of low anti-viral IFN response [81]. Besides, the initial inflammatory burst would mobilize dendritic cells that initiate adaptive immunity and promote the production of antiviral IgG presenting pathogenic glycomes in some individuals [99]. The resulting immune complexes would further activate the inflammatory response of lung macrophages having upregulated FcγR in this inflammatory context, a mechanism associated with severity [100, 101]. The production of virions by AT2s would also stimulate the highly responsive MoMacs located in their vicinity. These complex synergistic responses in predisposed individuals would then lead to uncontrolled inflammation, pneumonia and acute respiratory disease syndrome, further complicated by the establishment of a fibrotic phase [76]. The infection of endothelial cells [102] and the pro-coagulatory inflammation [103] would further conduct a thrombotic phenomenon and dissemination of the infection, leading to the systemic cytokinic storm of COVID-19. Consequently, this proposed model suggests that the identification of the molecular components involved in the initial viral interaction with monocytes/macrophages, particularly with the MoMacs that appeared most inflammatory, is of utmost importance, in order to develop effective interfering prophylactic strategies.

### Supplementary Information

Below is the link to the electronic supplementary material.

Supplementary file1 (XLSX 11 KB)

Supplementary file2 (PPTX 8514 KB)

Supplementary file3 ElisaIgG (PPTX 40 KB)

Supplementary file4 (DOCX 236 KB)

Supplementary file5 (DOCX 19 KB)

Supplementary file6 (PPTX 541 KB)

Supplementary file7 (PPTX 348 KB)

Supplementary file8 (XLSX 41 KB)

Supplementary file9 (XLSX 121 KB)

Supplementary file10 (PPTX 46 KB)

Supplementary file11 (XLSX 13 KB)

Supplementary file12 (XLSX 15 KB)

Supplementary file13 (PPTX 6248 KB)

Supplementary file14 (PPTX 142 KB)

Supplementary file15 (PPTX 86 KB)

Supplementary file16 (XLSX 21 KB)

Supplementary file17 (DOCX 24 KB)

Supplementary file18 (DOCX 24 KB)

Supplementary file19 (DOCX 25 KB)

Supplementary file20 (DOCX 22 KB)

## Data Availability

The output files obtained from the Cell Ranger analysis have been deposited on the Gene Expression Omnibus repository of the NCBI. The control lung data are available under https://www.ncbi.nlm.nih.gov/geo/query/acc.cgi?acc=GSE218788; of note, the donors 1 and 2 of this record correspond to donors 4 and 5 in this study. The infected lung data have been deposited under https://www.ncbi.nlm.nih.gov/geo/query/acc.cgi?acc=GSE246128. The raw data from the scRNA-seq (fastq files) cannot be placed on a public repository due to legal issues. Indeed, the data were obtained from anonymized donors under the approvement of the Agence de la Biomédecine and by the Ministère de l’éducation nationale, de l’Enseignement Supérieur et de la Recherche, Direction Générale de la Recherche et de l’Innovation and raw RNA-seq data may possibly permit identification of donor (10.15252/embr.201948316), therefore conflicting with the respect of anonymization. No novel original codes were developed in this study. The codes used, which are classically used by analysts of scRNA-seq data, are available from the corresponding authors upon request. Further information and requests for resources and reagents should be directed to and will be fulfilled by the corresponding authors.

## Abbreviations

AMs: Alveolar macrophages
MoMacs: Monocyte-derived macrophages
cMos: Classical monocytes
ncMos: Non-classical monocytes
EVLP: Ex vivo lung perfusion
scRNA-seq: Single cell RNA sequencing
C: Cluster
DEGs: Differentially expressed genes
