# Supplementary material for: SARS-CoV2 infection in whole lung primarily targets macrophages that display subset-specific responses
Source: Cell Mol Life Sci. 2024 Aug 15;81(1):351. doi: 10.1007/s00018-024-05322-z (PMC11335275; doi:10.1007/s00018-024-05322-z)
Supplement: Supplementary file 2 — Supplementary file2 (PPTX 8514 KB) [file 18_2024_5322_MOESM2_ESM.pptx]

## Slide 1
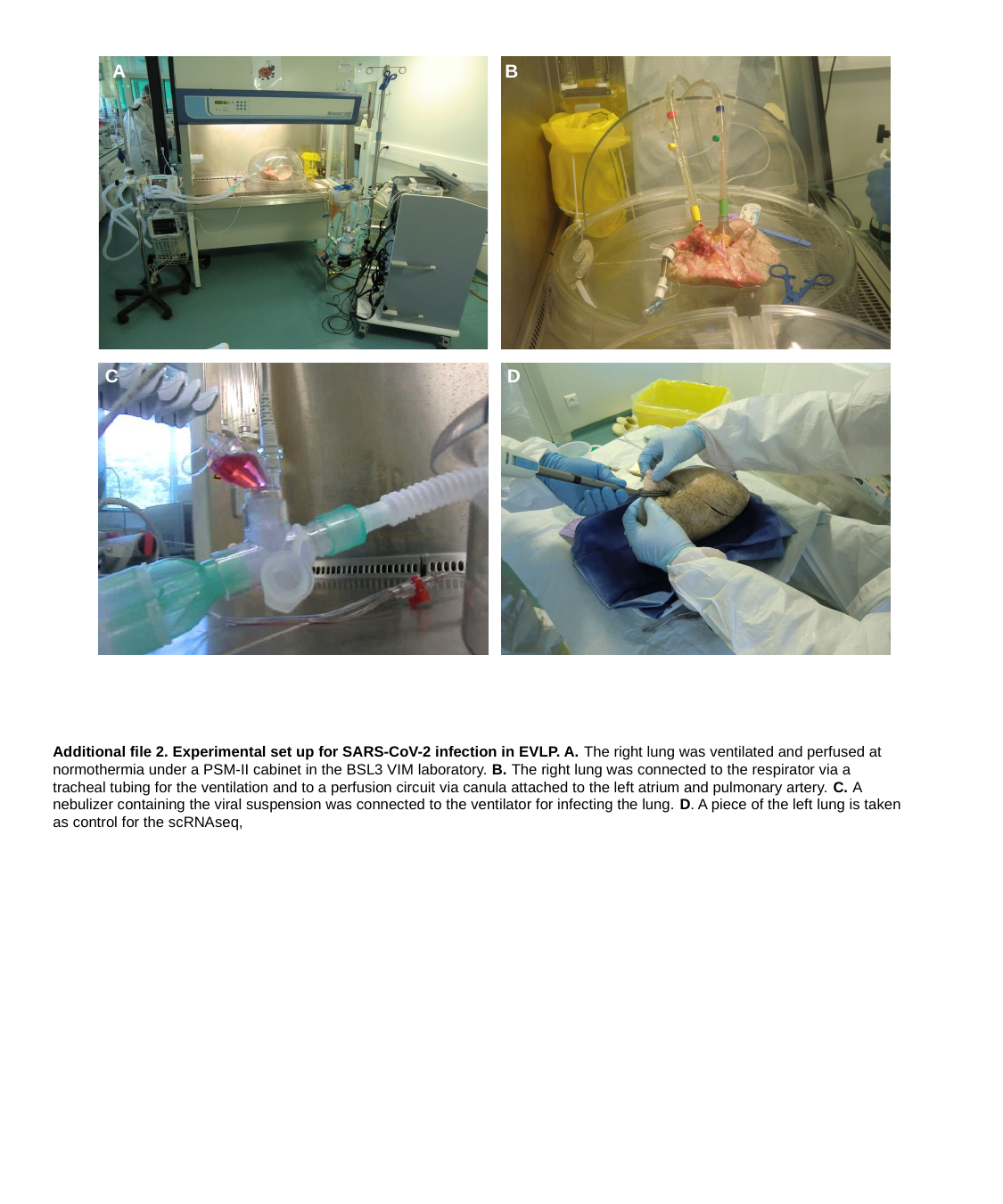

B
A
C
D
Additional file 2. Experimental set up for SARS-CoV-2 infection in EVLP. A. The right lung was ventilated and perfused at normothermia under a PSM-II cabinet in the BSL3 VIM laboratory. B. The right lung was connected to the respirator via a tracheal tubing for the ventilation and to a perfusion circuit via canula attached to the left atrium and pulmonary artery. C. A nebulizer containing the viral suspension was connected to the ventilator for infecting the lung. D. A piece of the left lung is taken as control for the scRNAseq,
