# Supplementary material for: SARS-CoV2 infection in whole lung primarily targets macrophages that display subset-specific responses
Source: Cell Mol Life Sci. 2024 Aug 15;81(1):351. doi: 10.1007/s00018-024-05322-z (PMC11335275; doi:10.1007/s00018-024-05322-z)
Supplement: Supplementary file 3 — Supplementary file3 ElisaIgG (PPTX 40 KB) [file 18_2024_5322_MOESM3_ESM.pptx]

## Slide 1
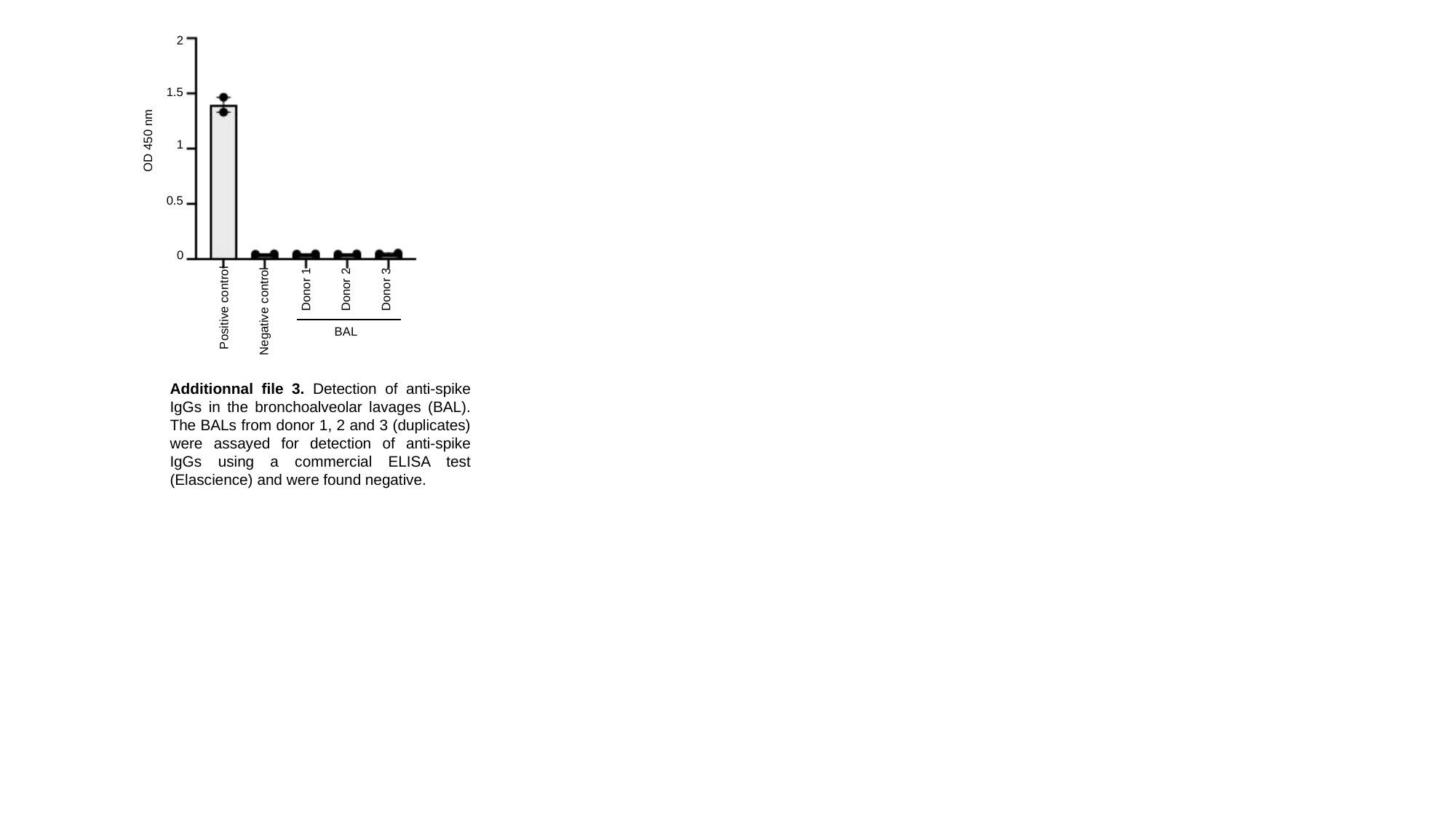

2
1.5
OD 450 nm
1
0.5
0
Donor 3
Donor 1
Donor 2
Positive control
Negative control
BAL
Additionnal file 3. Detection of anti-spike IgGs in the bronchoalveolar lavages (BAL). The BALs from donor 1, 2 and 3 (duplicates) were assayed for detection of anti-spike IgGs using a commercial ELISA test (Elascience) and were found negative.
