# Supplementary material for: SARS-CoV2 infection in whole lung primarily targets macrophages that display subset-specific responses
Source: Cell Mol Life Sci. 2024 Aug 15;81(1):351. doi: 10.1007/s00018-024-05322-z (PMC11335275; doi:10.1007/s00018-024-05322-z)
Supplement: Supplementary file 5 — Supplementary file5 (DOCX 19 KB) [file 18_2024_5322_MOESM5_ESM.docx]

**Additional file 5. Filtration results of the scRNA-seq samples (resulting cell numbers).** The table lists the cell samples used in scRNA-seq from 5 donors at T0 (before EVLP) and at T10 (10 h of EVLP with or without virus) as indicated in the description column. The resulting cell numbers after each scRNA-seq processing step are reported.

| **scRNA-seq samples** | **description** | **#cells before Scrublet** | **#cells after**  **Scrublet** | **#cells post mitochondrial gene cut-off** | **#cells post mitochondrial gene cut-off** | **#cells post filtration on azimuth score (>0.6)** | **#cells post filtration on minimal representation (>10%) in clusters** |
| --- | --- | --- | --- | --- | --- | --- | --- |
| **Donor 1-T0** | No EVLP | 9391 | 9271 | 5991 | 15808 | 12492 | 11240 |
| **Donor 1-T10** | 10 h EVLP + WL SARS-CoV-2 | 13737 | 13736 | 9817 |  |  |  |
| **Donor 2-T0** | No EVLP | 11052 | 11052 | 7110 | 26750 | 23267 | 21708 |
| **Donor 2-T10** | 10 h EVLP + D614G-a SARS-CoV-2 | 8354 | 8354 | 5640 |  |  |  |
| **Donor 2-T0-MHC2** | No EVLP & HLA-DR+ cell enrichment | 17163 | 16070 | 11766 |  |  |  |
| **Donor 2-T10-MHC2** | 10 h EVLP + D614G-a SARS-CoV-2 & HLA-DR+ cell enrichment | 6048 | 3738 | 2234 |  |  |  |
| **Donor 3-T0** | No EVLP | 6238 | 6237 | 2782 | 12911 | 11405 | 10793 |
| **Donor 3-T10** | 10 h EVLP + D614G-b SARS-CoV-2 | 5739 | 5644 | 3840 |  |  |  |
| **Donor 3-T0-MHC2** | No EVLP & HLA-DR+ cell enrichment | 5330 | 5251 | 3040 |  |  |  |
| **Donor 3-T10-MHC2** | 10 h EVLP + D614G-b SARS-CoV-2& HLA-DR+ cell enrichment | 4602 | 4601 | 3249 |  |  |  |
| **Donor 4-T0** | No EVLP | 8288 | 8152 | 6116 | 14581 | 12024 | 11142 |
| **Donor 4-T10** | 10 h EVLP | 20873 | 20867 | 8465 |  |  |  |
| **Donor 5-T0** | No EVLP | 12120 | 12118 | 7383 | 16203 | 12931 | 11854 |
| **Donor 5-T10** | 10 h EVLP | 12918 | 12588 | 8820 |  |  |  |
| **Integrated samples** |  |  |  | 86253 | 86253 | 72119 | 66737 |
