# Supplementary material for: SARS-CoV2 infection in whole lung primarily targets macrophages that display subset-specific responses
Source: Cell Mol Life Sci. 2024 Aug 15;81(1):351. doi: 10.1007/s00018-024-05322-z (PMC11335275; doi:10.1007/s00018-024-05322-z)
Supplement: Supplementary file 6 — Supplementary file6 (PPTX 541 KB) [file 18_2024_5322_MOESM6_ESM.pptx]

## Slide 1
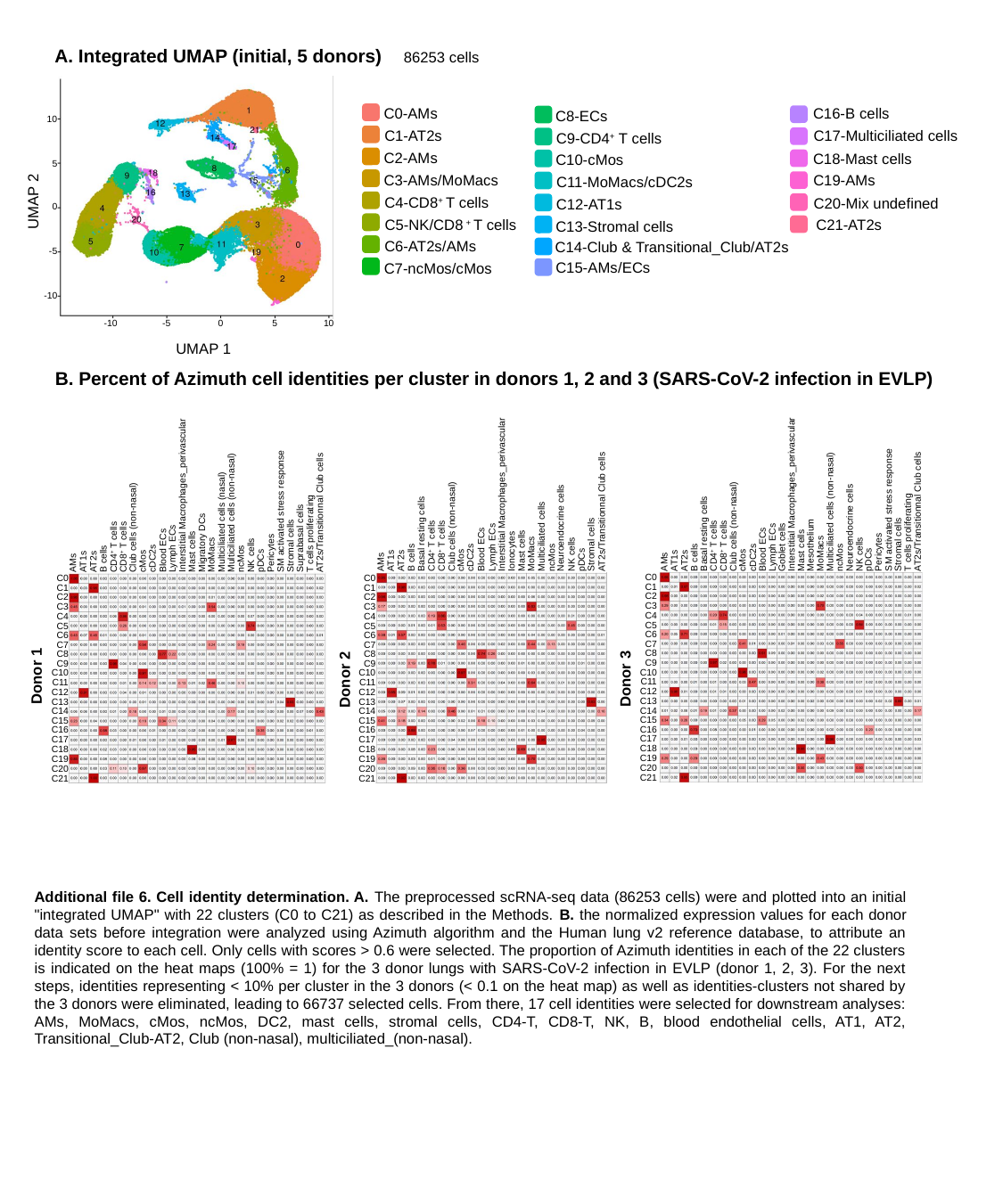

A. Integrated UMAP (initial, 5 donors)
86253 cells
C0-AMs
C16-B cells
C8-ECs
10
C17-Multiciliated cells
C1-AT2s
C9-CD4+ T cells
C2-AMs
C18-Mast cells
C10-cMos
5
C19-AMs
C3-AMs/MoMacs
C11-MoMacs/cDC2s
UMAP 2
C4-CD8+ T cells
C20-Mix undefined
C12-AT1s
0
C21-AT2s
C5-NK/CD8 + T cells
C13-Stromal cells
C6-AT2s/AMs
C14-Club & Transitional_Club/AT2s
-5
C15-AMs/ECs
C7-ncMos/cMos
-10
-10
-5
0
5
10
UMAP 1
B. Percent of Azimuth cell identities per cluster in donors 1, 2 and 3 (SARS-CoV-2 infection in EVLP)
AT2s/Transitionnal Club cells
Neuroendocrine cells
NK cells
pDCs
Stromal cells
Blood ECs
Lymph ECs
Club cells (non-nasal)
Basal resting cells
Multiciliated cells
ncMos
CD4+ T cells
CD8+ T cells
Ionocytes
Mast cells
MoMacs
B cells
cMos
cDC2s
AMs
AT1s
AT2s
Interstitial Macrophages_perivascular
AT2s/Transitionnal Club cells
SM activated stress response
Multiciliated cells (non-nasal)
Multiciliated cells (nasal)
NK cells
pDCs
Stromal cells
Suprabasal cells
T cells proliferating
Blood ECs
Lymph ECs
Club cells (non-nasal)
Pericytes
ncMos
Migratory DCs
CD4+ T cells
CD8+ T cells
Mast cells
MoMacs
B cells
cMos
cDC2s
AMs
AT1s
AT2s
Interstitial Macrophages_perivascular
Interstitial Macrophages_perivascular
AT2s/Transitionnal Club cells
SM activated stress response
Multiciliated cells (non-nasal)
Neuroendocrine cells
NK cells
pDCs
Stromal cells
T cells proliferating
Blood ECs
Lymph ECs
Goblet cells
Club cells (non-nasal)
Basal resting cells
Pericytes
ncMos
Mesothelium
CD4+ T cells
CD8+ T cells
Mast cells
MoMacs
B cells
cMos
cDC2s
AMs
AT1s
AT2s
C0
C1
C2
C3
C4
C5
C6
C7
C8
C9
C10
C11
C12
C13
C14
C15
C16
C17
C18
C19
C20
C21
C0
C1
C2
C3
C4
C5
C6
C7
C8
C9
C10
C11
C12
C13
C14
C15
C16
C17
C18
C19
C20
C21
C0
C1
C2
C3
C4
C5
C6
C7
C8
C9
C10
C11
C12
C13
C14
C15
C16
C17
C18
C19
C20
C21
Donor 1
Donor 3
Donor 2
Additional file 6. Cell identity determination. A. The preprocessed scRNA-seq data (86253 cells) were and plotted into an initial "integrated UMAP" with 22 clusters (C0 to C21) as described in the Methods. B. the normalized expression values for each donor data sets before integration were analyzed using Azimuth algorithm and the Human lung v2 reference database, to attribute an identity score to each cell. Only cells with scores > 0.6 were selected. The proportion of Azimuth identities in each of the 22 clusters is indicated on the heat maps (100% = 1) for the 3 donor lungs with SARS-CoV-2 infection in EVLP (donor 1, 2, 3). For the next steps, identities representing < 10% per cluster in the 3 donors (< 0.1 on the heat map) as well as identities-clusters not shared by the 3 donors were eliminated, leading to 66737 selected cells. From there, 17 cell identities were selected for downstream analyses: AMs, MoMacs, cMos, ncMos, DC2, mast cells, stromal cells, CD4-T, CD8-T, NK, B, blood endothelial cells, AT1, AT2, Transitional_Club-AT2, Club (non-nasal), multiciliated_(non-nasal).
