# Supplementary material for: SARS-CoV2 infection in whole lung primarily targets macrophages that display subset-specific responses
Source: Cell Mol Life Sci. 2024 Aug 15;81(1):351. doi: 10.1007/s00018-024-05322-z (PMC11335275; doi:10.1007/s00018-024-05322-z)
Supplement: Supplementary file 7 — Supplementary file7 (PPTX 348 KB) [file 18_2024_5322_MOESM7_ESM.pptx]

## Slide 1
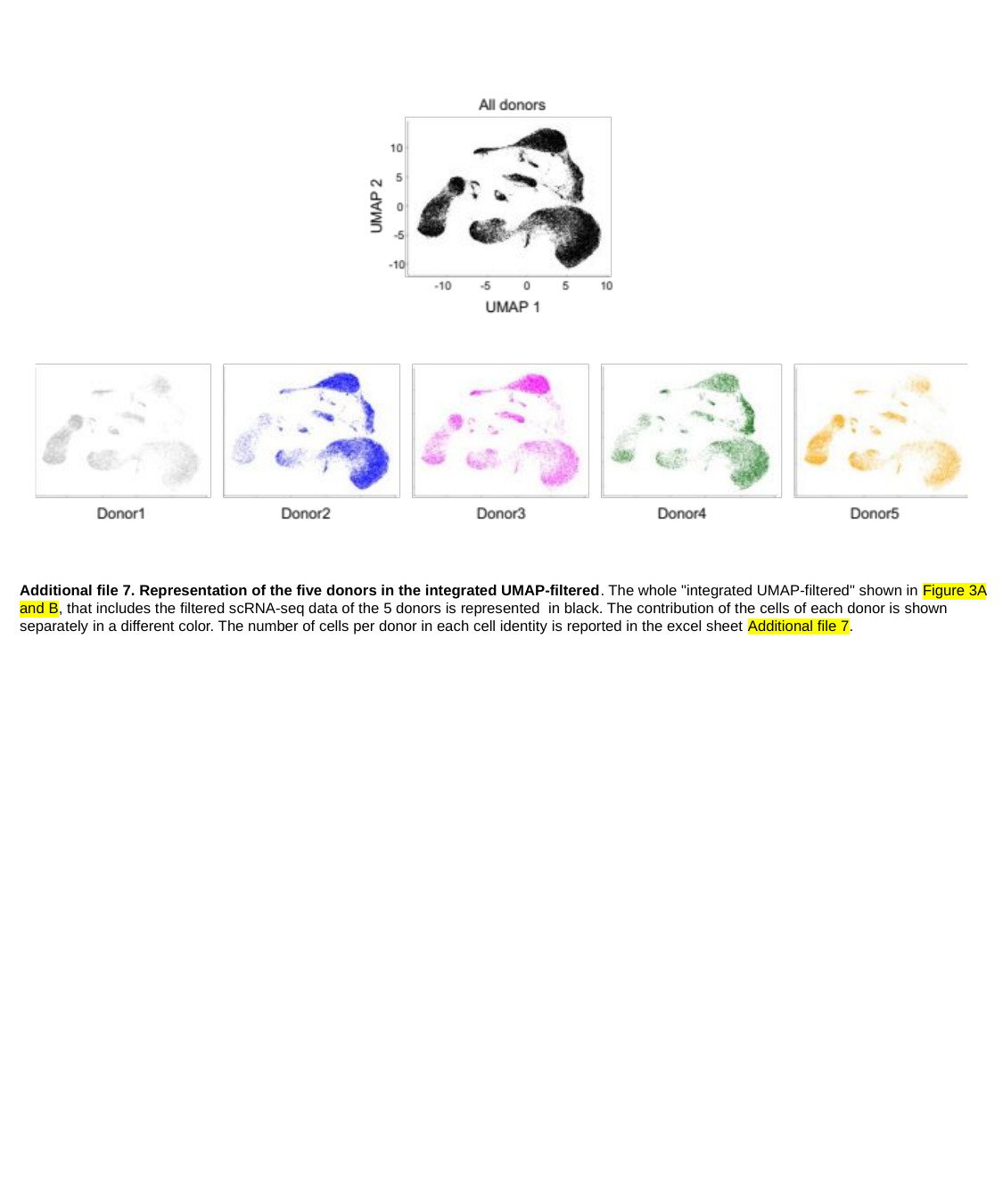

Additional file 7. Representation of the five donors in the integrated UMAP-filtered. The whole "integrated UMAP-filtered" shown in Figure 3A and B, that includes the filtered scRNA-seq data of the 5 donors is represented in black. The contribution of the cells of each donor is shown separately in a different color. The number of cells per donor in each cell identity is reported in the excel sheet Additional file 7.
