# Supplementary material for: SARS-CoV2 infection in whole lung primarily targets macrophages that display subset-specific responses
Source: Cell Mol Life Sci. 2024 Aug 15;81(1):351. doi: 10.1007/s00018-024-05322-z (PMC11335275; doi:10.1007/s00018-024-05322-z)
Supplement: Supplementary file 10 — Supplementary file10 (PPTX 46 KB) [file 18_2024_5322_MOESM10_ESM.pptx]

## Slide 1
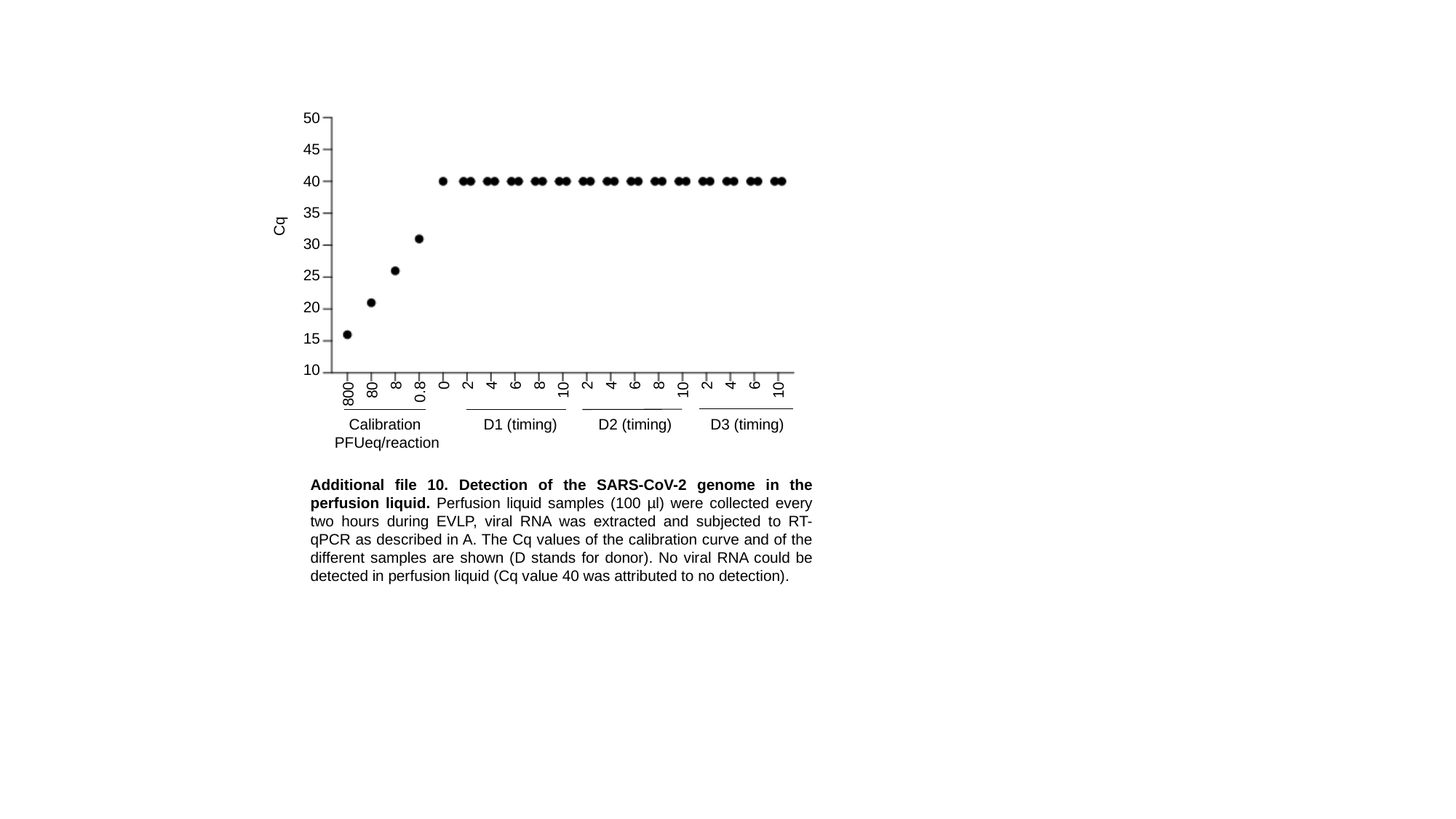

50
45
40
35
Cq
30
25
20
15
10
8
0
2
4
6
8
2
4
6
8
2
4
6
80
10
10
10
0.8
800
Calibration
PFUeq/reaction
D1 (timing)
D2 (timing)
D3 (timing)
Additional file 10. Detection of the SARS-CoV-2 genome in the perfusion liquid. Perfusion liquid samples (100 µl) were collected every two hours during EVLP, viral RNA was extracted and subjected to RT-qPCR as described in A. The Cq values of the calibration curve and of the different samples are shown (D stands for donor). No viral RNA could be detected in perfusion liquid (Cq value 40 was attributed to no detection).
