# Supplementary material for: SARS-CoV2 infection in whole lung primarily targets macrophages that display subset-specific responses
Source: Cell Mol Life Sci. 2024 Aug 15;81(1):351. doi: 10.1007/s00018-024-05322-z (PMC11335275; doi:10.1007/s00018-024-05322-z)
Supplement: Supplementary file 13 — Supplementary file13 (PPTX 6248 KB) [file 18_2024_5322_MOESM13_ESM.pptx]

## Slide 1
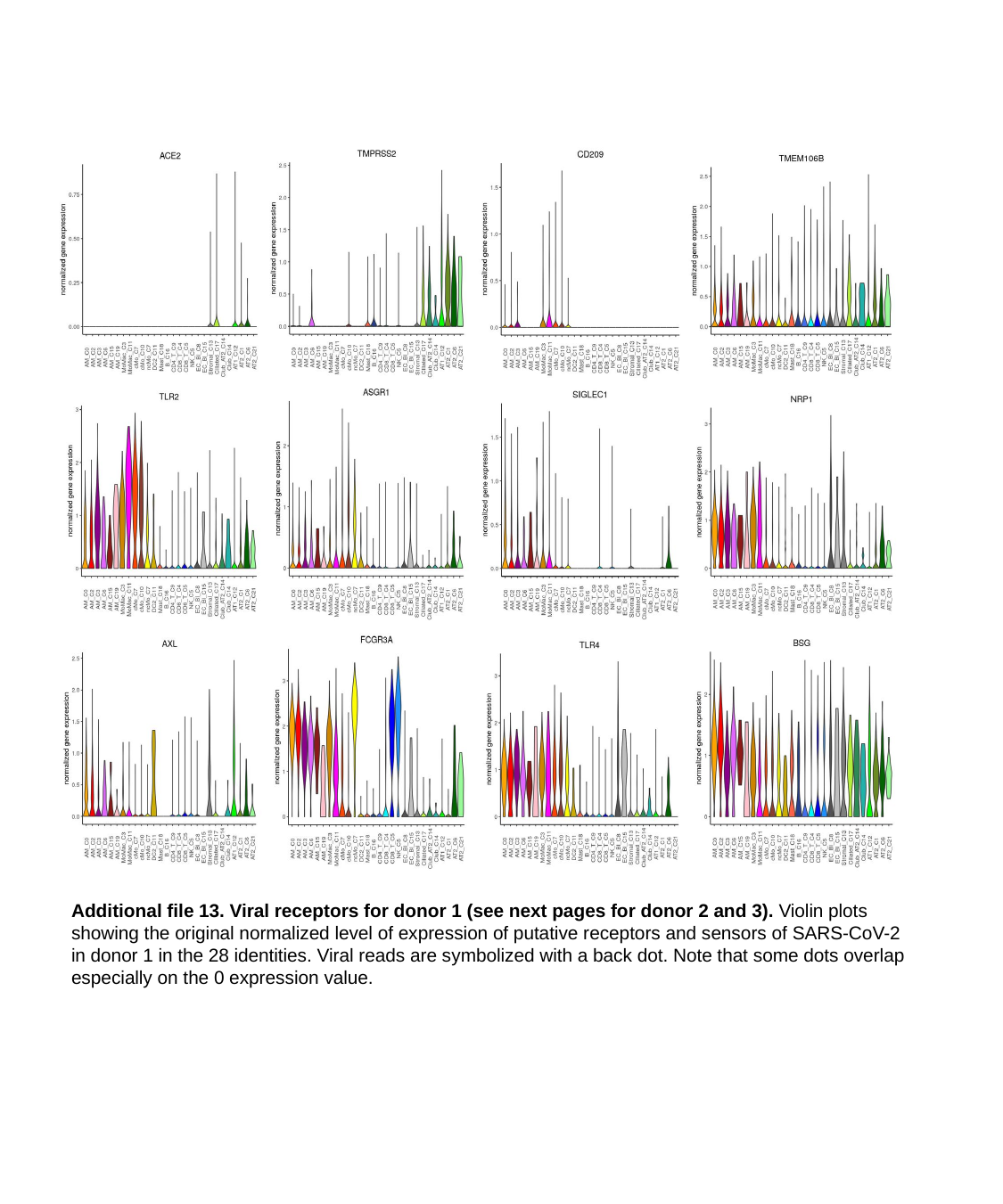

Additional file 13. Viral receptors for donor 1 (see next pages for donor 2 and 3). Violin plots showing the original normalized level of expression of putative receptors and sensors of SARS-CoV-2 in donor 1 in the 28 identities. Viral reads are symbolized with a back dot. Note that some dots overlap especially on the 0 expression value.

## Slide 2
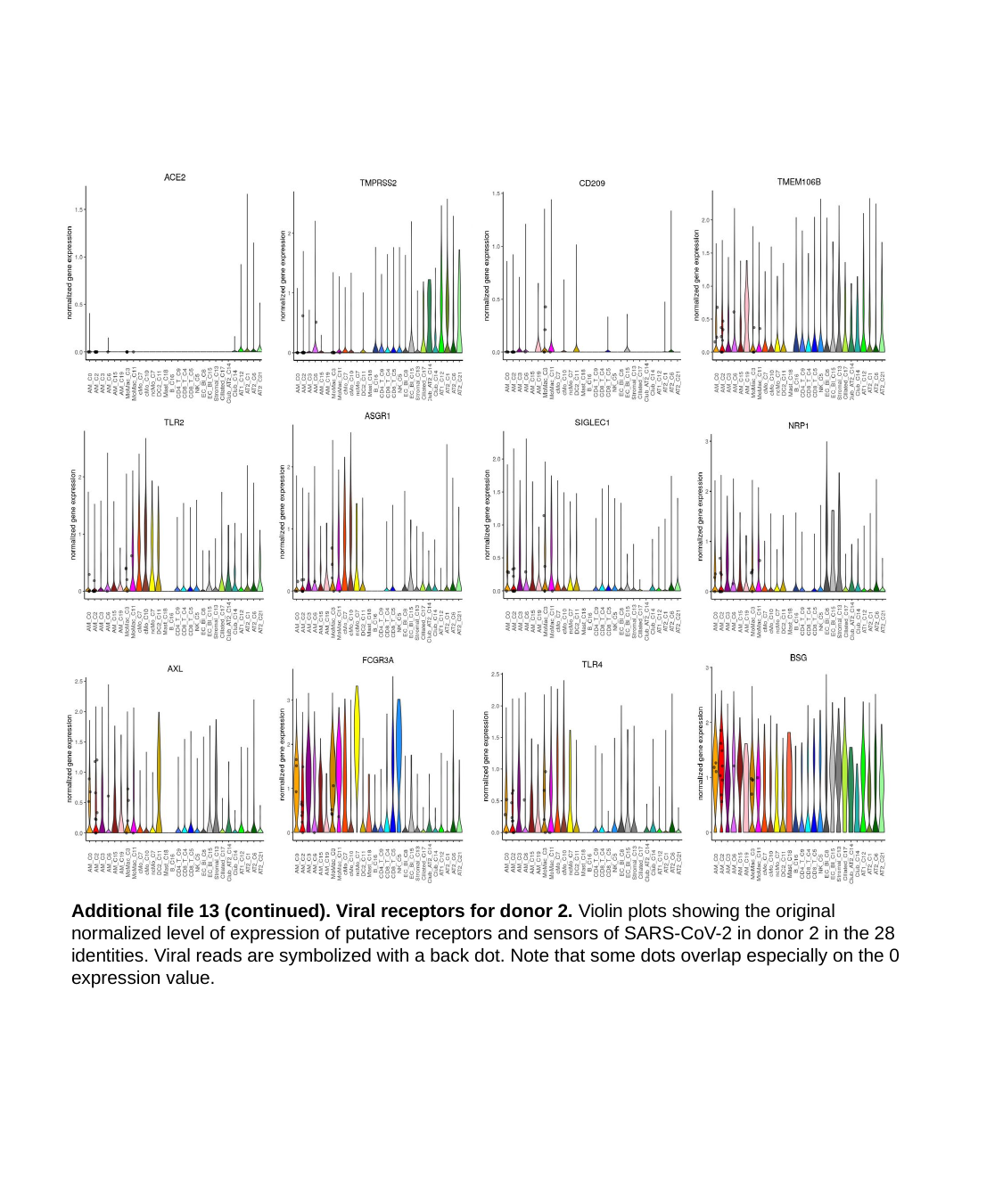

Additional file 13 (continued). Viral receptors for donor 2. Violin plots showing the original normalized level of expression of putative receptors and sensors of SARS-CoV-2 in donor 2 in the 28 identities. Viral reads are symbolized with a back dot. Note that some dots overlap especially on the 0 expression value.

## Slide 3
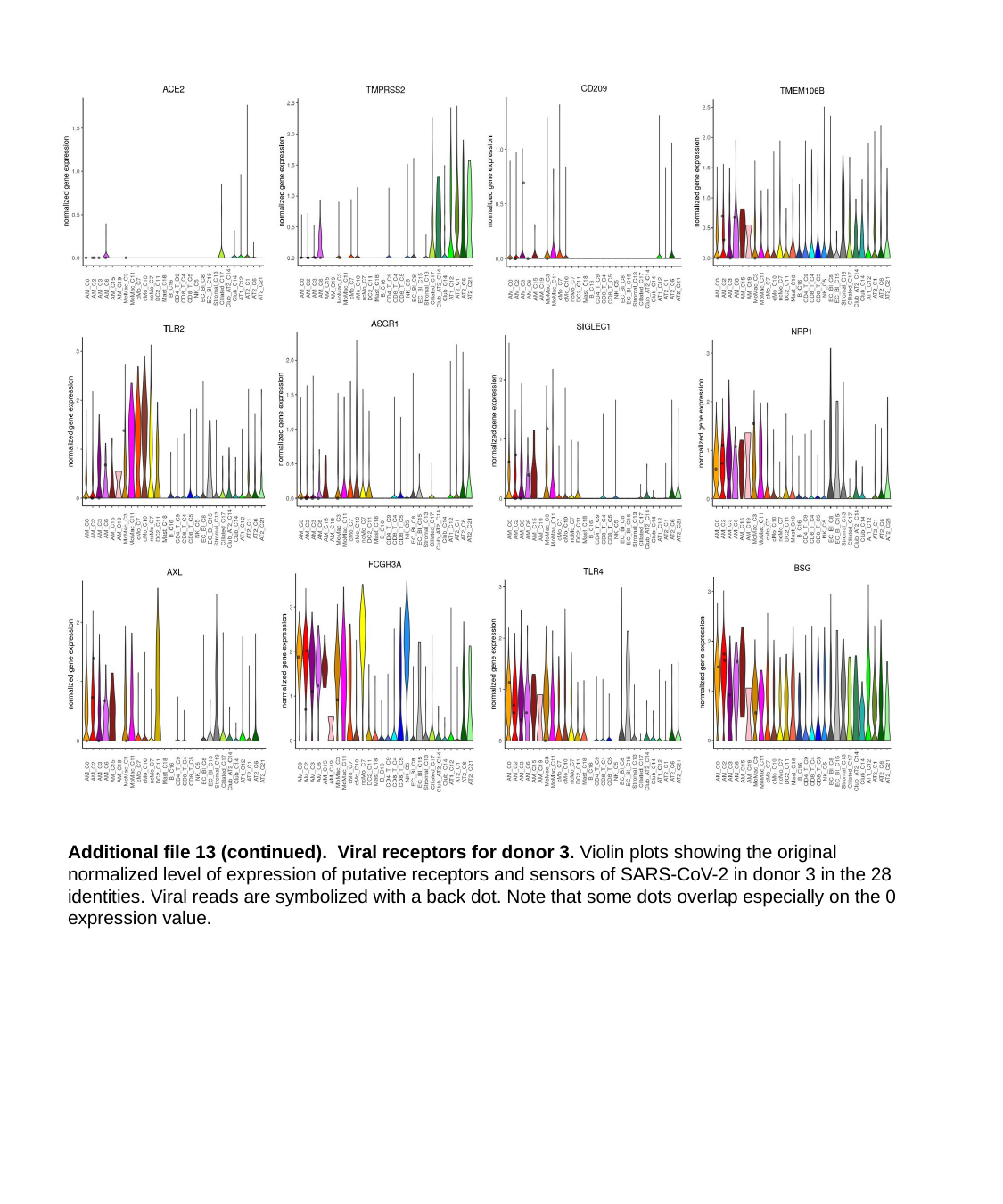

Additional file 13 (continued). Viral receptors for donor 3. Violin plots showing the original normalized level of expression of putative receptors and sensors of SARS-CoV-2 in donor 3 in the 28 identities. Viral reads are symbolized with a back dot. Note that some dots overlap especially on the 0 expression value.
