# Supplementary material for: SARS-CoV2 infection in whole lung primarily targets macrophages that display subset-specific responses
Source: Cell Mol Life Sci. 2024 Aug 15;81(1):351. doi: 10.1007/s00018-024-05322-z (PMC11335275; doi:10.1007/s00018-024-05322-z)
Supplement: Supplementary file 14 — Supplementary file14 (PPTX 142 KB) [file 18_2024_5322_MOESM14_ESM.pptx]

## Slide 1
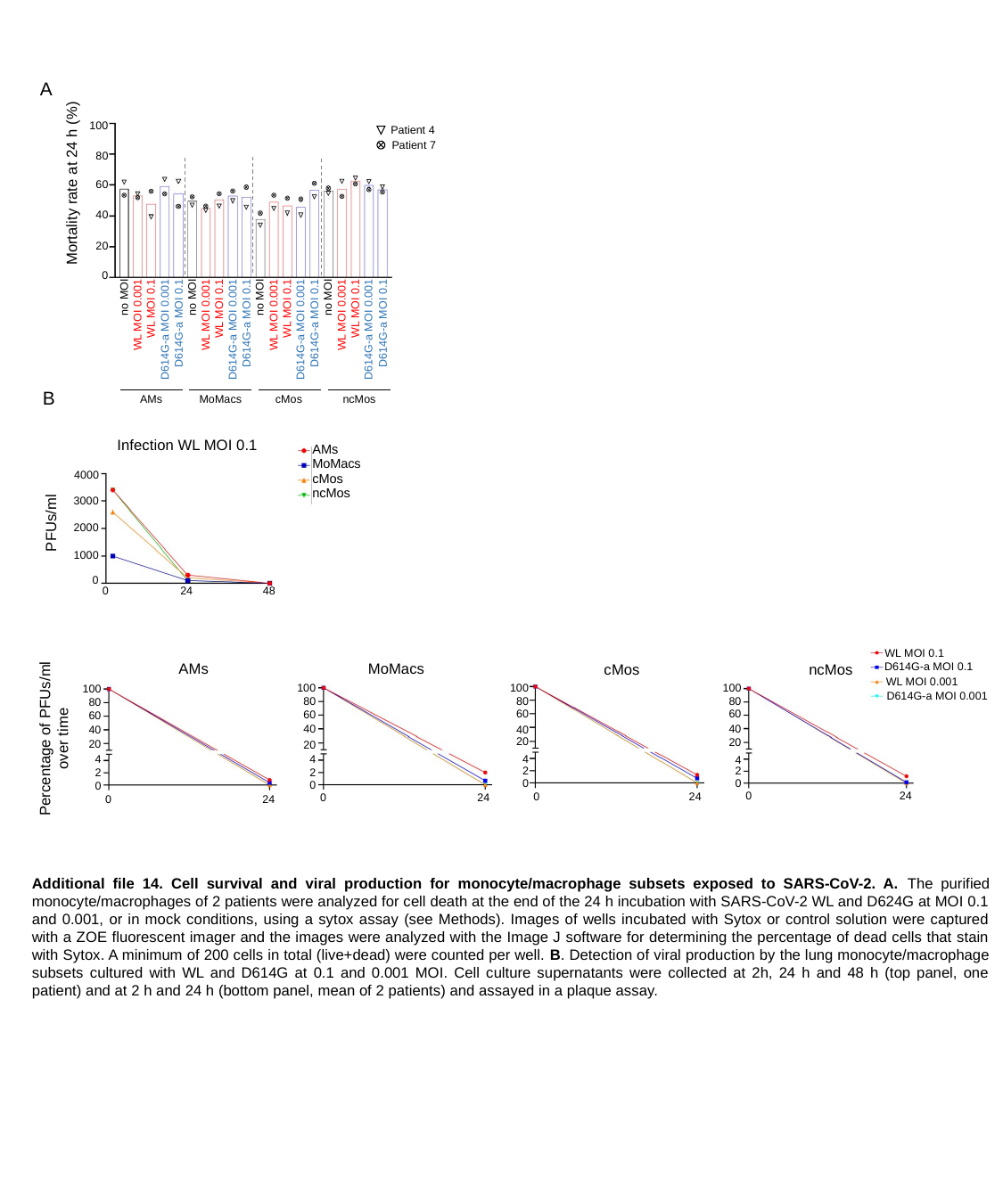

A
100
Patient 4
Patient 7
80
Mortality rate at 24 h (%)
60
40
20
0
no MOI
no MOI
no MOI
no MOI
WL MOI 0.1
WL MOI 0.1
 WL MOI 0.1
D614G-a MOI 0.1
D614G-a MOI 0.1
WL MOI 0.001
WL MOI 0.1
WL MOI 0.001
WL MOI 0.001
D614G-a MOI 0.1
WL MOI 0.001
D614G-a MOI 0.001
D614G-a MOI 0.001
D614G-a MOI 0.001
D614G-a MOI 0.1
D614G-a MOI 0.001
B
AMs
MoMacs
cMos
ncMos
Infection WL MOI 0.1
AMs
MoMacs
4000
cMos
ncMos
3000
PFUs/ml
2000
1000
0
24
48
0
WL MOI 0.1
AMs
MoMacs
D614G-a MOI 0.1
cMos
ncMos
WL MOI 0.001
100
100
100
100
D614G-a MOI 0.001
80
80
80
80
60
60
60
60
Percentage of PFUs/ml over time
40
40
40
40
20
20
20
20
4
4
4
4
2
2
2
2
0
0
0
0
0
24
0
24
0
24
0
24
Additional file 14. Cell survival and viral production for monocyte/macrophage subsets exposed to SARS-CoV-2. A. The purified monocyte/macrophages of 2 patients were analyzed for cell death at the end of the 24 h incubation with SARS-CoV-2 WL and D624G at MOI 0.1 and 0.001, or in mock conditions, using a sytox assay (see Methods). Images of wells incubated with Sytox or control solution were captured with a ZOE fluorescent imager and the images were analyzed with the Image J software for determining the percentage of dead cells that stain with Sytox. A minimum of 200 cells in total (live+dead) were counted per well. B. Detection of viral production by the lung monocyte/macrophage subsets cultured with WL and D614G at 0.1 and 0.001 MOI. Cell culture supernatants were collected at 2h, 24 h and 48 h (top panel, one patient) and at 2 h and 24 h (bottom panel, mean of 2 patients) and assayed in a plaque assay.
