# Supplementary material for: SARS-CoV2 infection in whole lung primarily targets macrophages that display subset-specific responses
Source: Cell Mol Life Sci. 2024 Aug 15;81(1):351. doi: 10.1007/s00018-024-05322-z (PMC11335275; doi:10.1007/s00018-024-05322-z)
Supplement: Supplementary file 15 — Supplementary file15 (PPTX 86 KB) [file 18_2024_5322_MOESM15_ESM.pptx]

## Slide 1
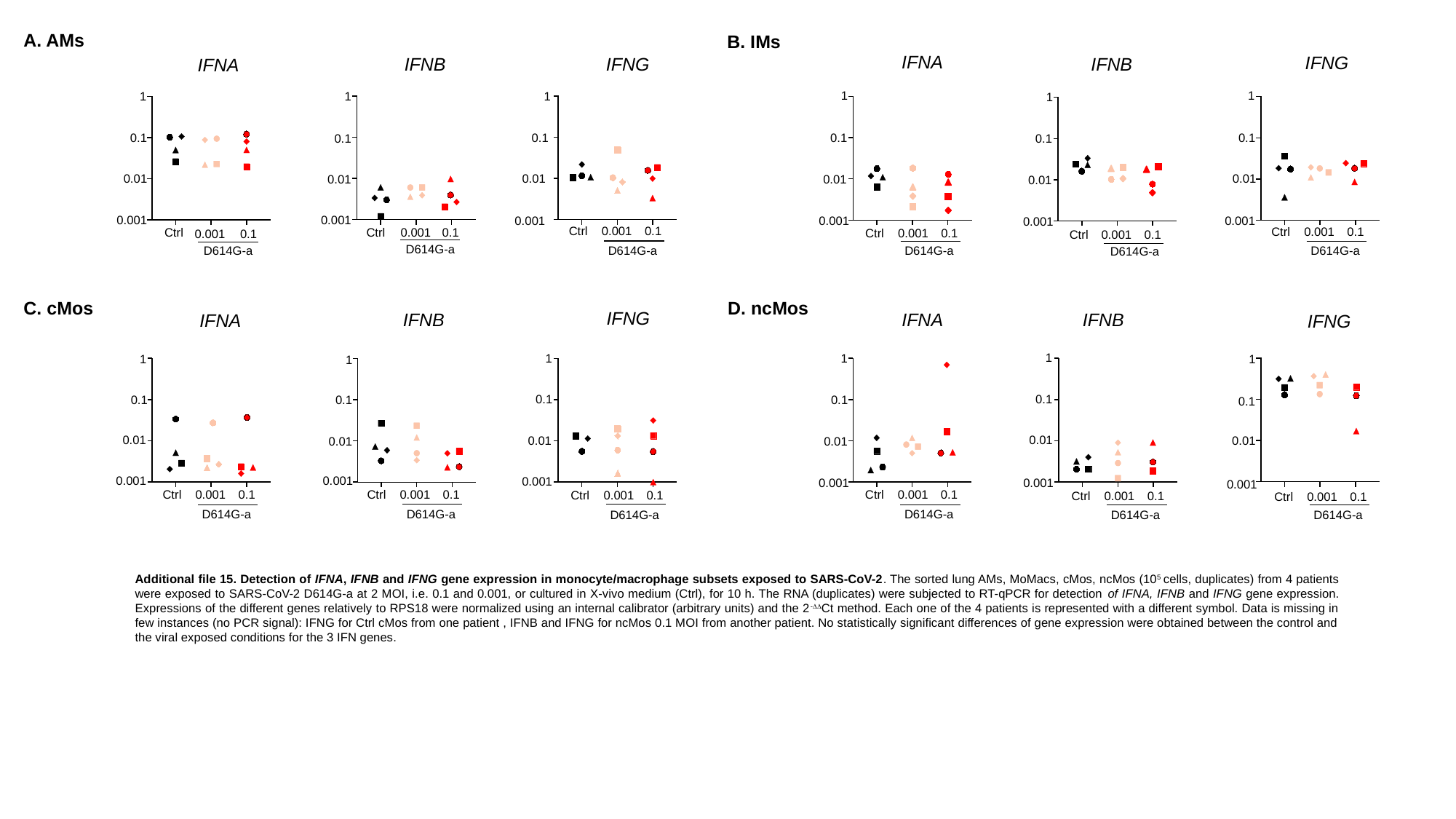

A. AMs
B. IMs
IFNA
IFNG
IFNB
1
0.1
0.01
0.001
Ctrl
0.001
0.1
D614G-a
IFNG
IFNB
IFNA
1
1
1
1
1
0.1
0.1
0.1
0.1
0.1
0.01
0.01
0.01
0.01
0.01
0.001
0.001
0.001
0.001
0.001
Ctrl
0.001
0.1
Ctrl
0.001
0.1
Ctrl
0.001
0.1
Ctrl
Ctrl
0.001
0.1
0.001
0.1
D614G-a
D614G-a
D614G-a
D614G-a
D614G-a
C. cMos
D. ncMos
IFNG
IFNB
IFNA
IFNB
IFNA
IFNG
1
1
1
0.1
0.01
0.001
1
0.1
0.01
0.001
1
0.1
0.01
0.001
1
0.1
0.1
0.1
0.01
0.01
0.01
0.001
0.001
0.001
Ctrl
0.001
0.1
Ctrl
0.001
0.1
D614G-a
Ctrl
0.001
0.1
D614G-a
Ctrl
0.001
0.1
D614G-a
Ctrl
0.001
0.1
D614G-a
Ctrl
0.001
0.1
D614G-a
D614G-a
Additional file 15. Detection of IFNA, IFNB and IFNG gene expression in monocyte/macrophage subsets exposed to SARS-CoV-2. The sorted lung AMs, MoMacs, cMos, ncMos (105 cells, duplicates) from 4 patients were exposed to SARS-CoV-2 D614G-a at 2 MOI, i.e. 0.1 and 0.001, or cultured in X-vivo medium (Ctrl), for 10 h. The RNA (duplicates) were subjected to RT-qPCR for detection of IFNA, IFNB and IFNG gene expression. Expressions of the different genes relatively to RPS18 were normalized using an internal calibrator (arbitrary units) and the 2-DDCt method. Each one of the 4 patients is represented with a different symbol. Data is missing in few instances (no PCR signal): IFNG for Ctrl cMos from one patient , IFNB and IFNG for ncMos 0.1 MOI from another patient. No statistically significant differences of gene expression were obtained between the control and the viral exposed conditions for the 3 IFN genes.
