# Supplementary material for: SARS-CoV2 infection in whole lung primarily targets macrophages that display subset-specific responses
Source: Cell Mol Life Sci. 2024 Aug 15;81(1):351. doi: 10.1007/s00018-024-05322-z (PMC11335275; doi:10.1007/s00018-024-05322-z)
Supplement: Supplementary file 17 — Supplementary file17 (DOCX 24 KB) [file 18_2024_5322_MOESM17_ESM.docx]

| **cytokine** | Comparisons between cell subsets (p-values) | | | | | | |
| --- | --- | --- | --- | --- | --- | --- | --- |
|  | Virus | MoMacs vs  AMs | cMos  vs  AMs | MoMacs  vs  cMos | AMs  vs ncMos | MoMacs vs  ncMos | cMos  vs ncMos |
| **CCL3** | Wuhan 0.001 | 0.0709 | 0.2169 | 0.9193 | 0.0313 | 0.7396 | 0.7504 |
|  | Wuhan 0.1 | 0.0003 | 0.0007 | 0.7307 | 0.0001 | 0.1694 | 0.0360 |
|  | D614G-a 0.001 | 0.1321 | 0.0870 | 0.8782 | 0.1126 | 0.3517 | 0.1963 |
|  | D614G-a 0.1 | 0.6258 | 0.5362 | 0.9922 | 0.4262 | 0.2868 | 0.8498 |
| **CCL2** | Wuhan 0.001 | 0.3618 | 0.1999 | 0.4477 | 0.3805 | 0.5144 | 0.9546 |
|  | Wuhan 0.1 | 0.0545 | 0.0401 | 0.1209 | 0.8641 | 0.0781 | 0.0018 |
|  | D614G-a 0.001 | 0.0906 | 0.1117 | 0.8999 | 0.0781 | 0.3750 | 0.8405 |
|  | D614G-a 0.1 | 0.0107 | 0.0100 | 0.0614 | 0.0014 | 0.0003 | 0.0006 |
| **CXCL8** | Wuhan 0.001 | 0.2369 | 0.6315 | 0.5943 | 0.2180 | 0.3435 | 0.1687 |
|  | Wuhan 0.1 | 0.3750 | 0.0606 | 0.1809 | 0.3958 | 0.3916 | 0.0215 |
|  | D614G-a 0.001 | 0.1563 | 0.0676 | 0.1967 | 0.0313 | 0.3327 | 0.2185 |
|  | D614G-a 0.1 | 0.4688 | 0.2124 | 0.6186 | 0.2202 | 0.9375 | 0.7897 |
| **TNF-α** | Wuhan 0.001 | 0.0113 | 0.0035 | 0.0141 | 0.3387 | 0.0166 | 0.0012 |
|  | Wuhan 0.1 | 0.1960 | 0.0162 | 0.0395 | 0.0040 | 0.0007 | 0.00002 |
|  | D614G-a 0.001 | 0.0098 | 0.8351 | 0.1402 | 0.6812 | 0.1987 | 0.8508 |
|  | D614G-a 0.1 | 0.2969 | 0.2575 | 0.3586 | 0.5781 | 0.6307 | 0.4231 |
| **IL-6** | Wuhan 0.001 | 0.0587 | 0.0938 | 0.7722 | 0.4604 | 0.0105 | 0.0156 |
|  | Wuhan 0.1 | 0.1924 | 0.4777 | 0.7792 | 0.6226 | 0.1521 | 0.0684 |
|  | D614G-a 0.001 | 0.4501 | 0.5781 | 0.2946 | 0.4076 | 0.5781 | 0.1862 |
|  | D614G-a 0.1 | 0.1620 | 0.1047 | 0.3804 | 0.1135 | 0.0234 | 0.0323 |
| **IL-10** | Wuhan 0.001 | 0.0099 | 0.0113 | 0.7058 | 0.6036 | 0.0619 | 0.0623 |
|  | Wuhan 0.1 | 0.0313 | 0.0401 | 0.5729 | 0.0297 | 0.0003 | 0.0086 |
|  | D614G-a 0.001 | 0.2130 | 0.9504 | 0.0963 | 0.6497 | 0.5118 | 0.4932 |
|  | D614G 0.1 | 0.3197 | 0.9660 | 0.6258 | 0.1097 | 0.7103 | 0.5673 |
| **IL-1β** | Wuhan 0.001 | 0.3751 | 0.8760 | 0.7327 | 0.4375 | 0.2968 | 0.2968 |
|  | Wuhan 0.1 | 0.0090 | 0.0135 | 0.7772 | 0.0234 | 0.0045 | 0.0009 |
|  | D614G-a 0.001 | 0.3555 | 0.6236 | 0.1848 | 0.9174 | 0.5315 | 0.7817 |
|  | D614G-a 0.1 | 0.6391 | 0.7456 | 0.9758 | 0.6983 | 0.8099 | 0.9668 |
| **CCL4** | Wuhan 0.001 | 0.4955 | 0.1438 | 0.2402 | 0.3125 | 0.0816 | 0.0133 |
|  | Wuhan 0.1 | 0.7683 | 0.6194 | 0.6109 | 0.0299 | 0.0359 | 0.3512 |
|  | D614G-a 0.001 | 0.7852 | 0.6001 | 0.2969 | 0.9836 | 0.7731 | 0.6875 |
|  | D614G-a 0.1 | 0.5705 | 0.1384 | 0.2740 | 0.5016 | 0.9394 | 0.3456 |
| **IL-1RA** | Wuhan 0.001 | 0.8655 | 0.9558 | 0.9375 | 0.7027 | 0.4486 | 0.3930 |
|  | Wuhan 0.1 | 0.6875 | 1.0000 | 0.9863 | 0.8510 | 0.8796 | 0.7642 |
|  | D614G-a 0.001 | 0.5501 | 0.3732 | 0.6216 | 0.7180 | 0.8920 | 0.5912 |
|  | D614G-a 0.1 | 0.2187 | 0.0799 | 0.7964 | 0.6860 | 0.0699 | 0.4068 |
| **IL-18** | Wuhan 0.001 | 0.8971 | 0.3928 | 0.5111 | 0.5839 | 0.3654 | 0.3750 |
|  | Wuhan 0.1 | 0.3006 | 0.1991 | 0.2969 | 0.0591 | 0.6245 | 0.8125 |
|  | D614G-a 0.001 | 0.5808 | 0.7767 | 0.8976 | 0.1814 | 0.8851 | 0.9572 |
|  | D614G-a 0.1 | 0.7720 | 0.8796 | 0.8082 | 0.4051 | 0.7490 | 0.5117 |

**Additional file 17.** **Cytokine and chemokine expression fold changes induced by SARS-COV-2 stimulation. Statistical comparisons between cell subsets.** The levels of cytokine concentrations were measured with a 12-plex Luminex kit. Ratios between cytokine levels of wells cultured for 24 h with virus (Wuhan or D614G strain) at 0.1 and 0.001 MOI versus plain medium were calculated. When cytokine levels were found below the threshold of the kit, the minimal value detected by the multiplex was assigned. To compare the data, a paired bilateral t-test was used when the data passed the Shapiro normality test. For cases that did not pass the Shapiro normality test, a non-parametric Mann Whitney test was performed. To indicate statistically significant higher levels in the first hand of the comparison, deep orange was used to color the box when the p-value was < 0.05 and light orange when the p-value laid between 0.05 and 0.08. In the reverse case, bleu and light blue were used.
