# Supplementary material for: SARS-CoV2 infection in whole lung primarily targets macrophages that display subset-specific responses
Source: Cell Mol Life Sci. 2024 Aug 15;81(1):351. doi: 10.1007/s00018-024-05322-z (PMC11335275; doi:10.1007/s00018-024-05322-z)
Supplement: Supplementary file 19 — Supplementary file19 (DOCX 25 KB) [file 18_2024_5322_MOESM19_ESM.docx]

| **cytokine** | Comparisons between cell subsets (p-values) | | | | | | |
| --- | --- | --- | --- | --- | --- | --- | --- |
|  | Virus | MoMacs vs  AMs | cMos vs  AMs | MoMacs vs  cMos | AMs  vs ncMos | MoMacs vs  ncMos | cMos  vs ncMos |
| **CCL3** | Wuhan 0.001 | 0.6941 | 0.5116 | 0.7893 | 0.0749 | 0.4290 | 0.8065 |
|  | Wuhan 0.1 | 0.0037 | 0.0156 | 0.1814 | 0.0156 | 0.4227 | 0.1814 |
|  | D614G-a 0.001 | 0.4227 | 1.0000 | 0.4451 | 1.0000 | 0.3711 | 1.0000 |
|  | D614G-a 0.1 | 0.9674 | 0.7915 | 0.4808 | 0.0517 | 0.1814 | 0.2702 |
| **CCL2** | Wuhan 0.001 | 0.3263 | 0.7522 | 0.4036 | 0.0754 | 0.0469 | 0.0322 |
|  | Wuhan 0.1 | 0.8442 | 0.2687 | 0.4401 | 0.0170 | 0.2116 | 0.4235 |
|  | D614G-a 0.001 | 0.5741 | 0.6667 | 0.9719 | 0.0160 | 0.0427 | 0.1003 |
|  | D614G-a 0.1 | 0.1484 | 0.0313 | 0.0401 | 0.0437 | 0.6875 | 0.0313 |
| **CXCL8** | Wuhan 0.001 | 0.7451 | 0.8602 | 0.8384 | 0.0374 | 0.2740 | 0.0870 |
|  | Wuhan 0.1 | 0.5631 | 0.1563 | 0.2012 | 0.0477 | 0.4151 | 0.3192 |
|  | D614G-a 0.001 | 0.1604 | 0.5230 | 0.2615 | 0.0051 | 0.0099 | 0.1814 |
|  | D614G-a 0.1 | 0.4569 | 0.2776 | 1.0000 | 0.0592 | 0.2621 | 0.3703 |
| **TNF-α** | Wuhan 0.001 | 0.0008 | 0.0018 | 0.6635 | 0.6020 | 0.0016 | 0.0007 |
|  | Wuhan 0.1 | 0.0137 | 0.0171 | 0.5102 | 0.0012 | 0.0005 | 0.0003 |
|  | D614G-a 0.001 | 0.0104 | 0.5807 | 0.0120 | 0.6036 | 0.0126 | 0.4227 |
|  | D614G-a 0.1 | 0.0703 | 0.1814 | 0.6499 | 0.4227 | 0.1056 | 0.2012 |
| **IL-6** | Wuhan 0.001 | 0.0046 | 0.0077 | 0.5083 | 0.0153 | 0.0001 | 0.0017 |
|  | Wuhan 0.1 | 0.0078 | 0.0055 | 0.9579 | 0.0156 | 0.0015 | 0.0006 |
|  | D614G-a 0.001 | 0.0342 | 0.3213 | 0.1541 | 0.6572 | 0.0349 | 0.5839 |
|  | D614G-a 0.1 | 0.0342 | 1.0000 | 0.5896 | 0.2945 | 0.0360 | 0.1775 |
| **IL-10** | Wuhan 0.001 | 0.0240 | 0.1446 | 0.2641 | 1.0000 | 0.0290 | 0.0684 |
|  | Wuhan 0.1 | 0.0001 | 0.0994 | 0.0346 | 0.1447 | 0.0001 | 0.0249 |
|  | D614G-a 0.001 | 0.1056 | 1.0000 | 0.2012 | 1.0000 | 0.1056 | 1.0000 |
|  | D614G-a 0.1 | 0.4227 | 1.0000 | 1.0000 | 1.0000 | 0.5807 | 1.0000 |
| **IL-1β** | Wuhan 0.001 | 1.0000 | 0.3710 | 0.3011 | 1.0000 | 0.8457 | 0.2918 |
|  | Wuhan 0.1 | 0.0063 | 0.0070 | 0.6263 | 0.0487 | 0.0048 | 0.0030 |
|  | D614G-a 0.001 | 0.3710 | 0.3710 | 1.0000 | 1.0000 | 0.3710 | 0.3710 |
|  | D614G-a 0.1 | 1.0000 | 1.0000 | 1.0000 | 1.0000 | 1.0000 | 1.0000 |
| **CCL4** | Wuhan 0.001 | 0.0164 | 0.0004 | 0.0252 | 0.9498 | 0.0075 | 0.0014 |
|  | Wuhan 0.1 | 0.3884 | 0.7023 | 0.8267 | 0.0251 | 0.0302 | 0.0795 |
|  | D614G-a 0.001 | 0.1256 | 0.8551 | 0.0702 | 0.1663 | 0.0667 | 0.8551 |
|  | D614G-a 0.1 | 0.7464 | 0.7473 | 0.6592 | 0.2214 | 0.2991 | 0.6888 |
| **IL-1RA** | Wuhan 0.001 | 0.0710 | 0.1008 | 0.5781 | 0.2280 | 0.0156 | 0.0041 |
|  | Wuhan 0.1 | 0.0373 | 0.4373 | 0.9895 | 0.0118 | 0.0098 | 0.0162 |
|  | D614G-a 0.001 | 0.0640 | 0.6415 | 0.1657 | 0.4895 | 0.0043 | 0.2476 |
|  | D614G-a 0.1 | 0.8951 | 0.2432 | 0.6376 | 0.1229 | 0.0161 | 0.2318 |
| **IL-18** | Wuhan 0.001 | 0.0591 | 0.0263 | 0.9405 | 1.0000 | 0.0591 | 0.0274 |
|  | Wuhan 0.1 | 0.6027 | 0.4443 | 0.1206 | 0.0448 | 0.0935 | 0.5839 |
|  | D614G-a 0.001 | 0.0137 | 0.0845 | 0.4162 | 0.3711 | 0.0466 | 0.2096 |
|  | D614G-a 0.1 | 0.2605 | 0.4227 | 0.7123 | 0.7893 | 0.1003 | 0.3711 |

**Additional file 19. Cytokine and chemokine net production induced by SARS-COV-2 stimulation. Statistical comparisons between cell subsets.** The levels of cytokine concentrations were measured with a 12-plex Luminex kit. Differences between cytokine levels of wells cultured for 24 h with virus (Wuhan or D614G strain) at 0.1 and 0.001 MOI minus plain medium were calculated. When cytokine levels were found below the threshold of the kit, a zero value was assigned. To compare the data, a paired bilateral t-test was used when the data passed the Shapiro normality test. For cases that did not pass the Shapiro normality test, a non-parametric Mann Whitney test was performed. To indicate statistically significant higher levels in the first hand of the comparison, deep orange was used to color the box when the p-value was < 0.05 and light orange when the p-value laid between 0.05 and 0.08. In the reverse case, bleu and light blue were used.
