# Supplementary material for: SARS-CoV2 infection in whole lung primarily targets macrophages that display subset-specific responses
Source: Cell Mol Life Sci. 2024 Aug 15;81(1):351. doi: 10.1007/s00018-024-05322-z (PMC11335275; doi:10.1007/s00018-024-05322-z)
Supplement: Supplementary file 20 — Supplementary file20 (DOCX 22 KB) [file 18_2024_5322_MOESM20_ESM.docx]

| **cytokine** | Comparisons between virus conditions (p-values) | | | | |
| --- | --- | --- | --- | --- | --- |
|  | Cell subsets | Wuhan  vs  D614G-a (0.001) | Wuhan  vs  D614G-a  (0.1) | Wuhan 0.1 vs  0.001 | D614G-a 0.1 vs  0.001 |
| **CCL3** | AMs | 0.0061 | 0.00003 | 0.0077 | 0.0505 |
|  | MoMacs | 0.1814 | 0.1814 | 0.7893 | 1.0000 |
|  | cMos | 0.3711 | 0.1350 | 0.3711 | 0.1814 |
|  | ncMos | 0.0682 | 0.3625 | 0.7387 | 1.0000 |
| **CCL2** | AMs | 0.3523 | 0.2211 | 0.0240 | 0.0018 |
|  | MoMacs | 0.0165 | 0.5936 | 0.9375 | 0.0591 |
|  | cMos | 0.1812 | 0.1176 | 0.6458 | 0.1814 |
|  | ncMos | 0.1814 | 0.0025 | 0.0138 | 0.00001 |
| **CXCL8** | AMs | 0.0377 | 0.0395 | 0.1775 | 0.6250 |
|  | MoMacs | 0.7406 | 0.0496 | 0.2012 | 0.2768 |
|  | cMos | 0.2012 | 1.0000 | 0.3613 | 1.0000 |
|  | ncMos | 0.0317 | 0.0066 | 0.0129 | 0.0749 |
| **TNF-α** | AMs | 0.1814 | 0.000004 | 0.0001 | 1.0000 |
|  | MoMacs | 0.0059 | 0.00002 | 0.0029 | 0.0971 |
|  | cMos | 0.0009 | 0.0008 | 0.0020 | 0.3884 |
|  | ncMos | 0.4227 | 0.1003 | 0.3613 | 1.0000 |
| **IL-6** | AMs | 0.0726 | 0.4924 | 0.0009 | 0.03603 |
|  | MoMacs | 0.0204 | 0.0746 | 0.0005 | 0.0184 |
|  | cMos | 0.0076 | 0.0313 | 0.0618 | 0.0283 |
|  | ncMos | 0.5592 | 0.0036 | 0.0081 | 0.0022 |
| **IL-10** | AMs | 1.0000 | 0.0729 | 0.0768 | 1.0000 |
|  | MoMacs | 0.1389 | 0.00003 | 0.0004 | 0.1003 |
|  | cMos | 0.0409 | 0.0309 | 0.0532 | 1.0000 |
|  | ncMos | 1.0000 | 1.0000 | 1.0000 | 1.0000 |
| **IL-1β** | AMs | 1.0000 | 0.0487 | 0.0963 | 1.0000 |
|  | MoMacs | 0.7089 | 0.0067 | 0.0045 | 0.6067 |
|  | cMos | 0.5838 | 0.0152 | 0.0012 | 1.0000 |
|  | ncMos | 1.0000 | 1.0000 | 1.0000 | 1.0000 |
| **CCL4** | AMs | 0.0167 | 0.0005 | 0.0006 | 0.3613 |
|  | MoMacs | 0.0653 | 0.0250 | 0.2969 | 0.3065 |
|  | cMos | 0.0003 | 0.0579 | 0.9375 | 0.7893 |
|  | ncMos | 0.0677 | 0.3659 | 0.5202 | 0.7643 |
| **IL-1RA** | AMs | 0.1647 | 0.9667 | 0.0938 | 0.1422 |
|  | MoMacs | 0.1094 | 0.4909 | 0.3750 | 0.6378 |
|  | cMos | 0.0360 | 0.3613 | 0.9994 | 0.9251 |
|  | ncMos | 1.0000 | 0.3082 | 0.7893 | 0.1003 |
| **IL-18** | AMs | 0.3711 | 0.0200 | 0.0621 | 0.7893 |
|  | MoMacs | 0.9968 | 0.4898 | 0.6750 | 0.5311 |
|  | cMos | 0.4259 | 1.0000 | 0.2807 | 0.4732 |
|  | ncMos | 1.0000 | 0.3711 | 0.3711 | 0.3711 |

**Additional file 20. Cytokine and chemokine net production induced by SARS-COV-2 stimulation. Statistical comparisons between virus condition (strains and virus doses).** The levels of cytokine concentrations were measured with a 12-plex Luminex kit. Differences between cytokine levels of wells cultured for 24 h with virus (Wuhan or D614G strain) at 0.1 and 0.001 MOI minus plain medium were calculated. When cytokine levels were found below the threshold of the kit, a zero value was assigned. To compare the data, a paired bilateral t-test was used when the data passed the Shapiro normality test. For cases that did not pass the Shapiro normality test, a non-parametric Mann Whitney test was performed. To indicate statistically significant higher levels in the first hand of the comparison, deep orange was used to color the box when the p-value was < 0.05 and light orange when the p-value laid between 0.05 and 0.08. In the reverse case, bleu and light blue were used.
